# Overexpression of HSF2 binding protein suppresses endoplasmic reticulum stress via regulating subcellular localization of CDC73 in hepatocytes

**DOI:** 10.1186/s13578-023-01010-w

**Published:** 2023-03-24

**Authors:** Jia Zhang, Tao Wang, Jianbin Bi, Mengyun Ke, Yifan Ren, Mengzhou Wang, Zhaoqing Du, Wuming Liu, Liangshuo Hu, Xiaogang Zhang, Xuemin Liu, Bo Wang, Zheng Wu, Yi Lv, Lingzhong Meng, Rongqian Wu

**Affiliations:** 1 National Local Joint Engineering Research Center for Precision Surgery & Regenerative Medicine, Shaanxi Provincial Center for Regenerative Medicine and Surgical Engineering, Center for Regenerative and Reconstructive Medicine, Med-X Institute, First Affiliated Hospital of Xi’an Jiaotong University, 124, 76 West Yanta Road, Xi’an, Shaanxi 710061 China; 2 Department of Gastroenterology, The Second Affiliated Hospital of Xi’an Jiaotong University, Xi’an, Shaanxi China; 3 Department of Hepatobiliary Surgery, First Affiliated Hospital of Xi’an Jiaotong University, Xi’an, Shaanxi China; 4 Department of Oncology, The Second Affiliated Hospital of Xi’an Jiaotong University, Xi’an, Shaanxi China; 5 Department of General Surgery, The Second Affiliated Hospital of Xi’an Jiaotong University, Xi’an, Shaanxi China; 6 Department of Hepatobiliary Surgery, Shaanxi Provincial People’s Hospital, Xi’an, Shaanxi China; 7 Anesthesiology and Perioperative Medicine, Mayo Clinic College of Medicine, Rochester, MN USA

**Keywords:** HSF2BP, ER stress, Hepatic I/R injury, CDC73

## Abstract

**Background:**

Endoplasmic reticulum (ER) stress plays an important role in the occurrence and development of various liver diseases. However, there are no effective prevention and treatment strategies. We aimed to determine the role of heat shock factor 2 binding protein (HSF2BP) in ER stress.

**Methods:**

HSF2BP expression in mice and cultured hepatocytes was measured during ER stress induced by tunicamycin, and its importance in ER stress was evaluated in hepatocyte-specific HSF2BP transgenic (TG) and knockout (KO) mice. The effects and mechanisms of HSF2BP on ER stress were further probed in hepatic ischemia-reperfusion (I/R) injury.

**Results:**

HSF2BP expression was significantly upregulated during tunicamycin-induced ER stress in mice and cultured hepatocytes. Liver injury and ER stress were reduced in HSF2BP overexpressing mice after treating with tunicamycin, but were aggravated in HSF2BP knockout mice compared to the controls. In hepatic I/R injury, HSF2BP expression was significantly upregulated, and HSF2BP overexpressing mice had reduced liver injury and inflammation. These improvements were associated with ER stress inhibition. However, these results were reversed in hepatocyte-specific HSF2BP knockout mice. HSF2BP overexpression increased cytoplasmic CDC73 levels and inhibited the JNK signaling pathway. CDC73 knockdown using siRNA eliminated the protection exerted by HSF2BP overexpression in hypoxia/reoxygenation (H/R)-induced ER stress in hepatocytes.

**Conclusion:**

HSF2BP is a previously uncharacterized regulatory factor in ER stress-likely acts by regulating CDC73 subcellular localization. The feasibility of HSF2BP-targeted treatment in ER stress-related liver disease deserves future research.

**Supplementary Information:**

The online version contains supplementary material available at 10.1186/s13578-023-01010-w.

## Introduction

The endoplasmic reticulum (ER) plays a vital role in the synthesis, folding and structural maturation of proteins in the cell. ER stress is a stress response triggered by multiple stimuli that disrupt the folding of proteins in the ER [1]. Hepatocytes are rich in ER, and ER stress is associated with many liver diseases, including alcoholic liver disease (ALD), nonalcoholic fatty liver disease, NAFLD), hepatic ischemia-reperfusion (I/R) injury, viral hepatitis, drug toxicity, and liver cancers [2]. However, there is no real effective target to relieve ER stress, and continuous efforts investigating novel targets to alleviate ER stress-related liver disease effectively are needed.

Heat shock factor 2 (HSF2) binding protein (HSF2BP) was first discovered in 1998 [3]. Subsequent studies revealed several biological functions involving HSF2BP, including spermatogenesis [3–7] and interaction with breast cancer-associated protein 2 to repair abundant meiotic DNA double-strand breaks [5, 6, 8, 9]. In addition, multiple studies have found a potential role of HSF2BP in a variety of diseases, such as multiple sclerosis [10], coronary artery disease [11], premature ovarian insufficiency [12] and lung adenocarcinoma [13]. Our recent study found that HSF2BP is protective in acute liver injury by regulating HSF2/HSP70/MAPK signaling [14]. However, the role of HSF2BP in other liver diseases has not been elucidated.

Cell division cycle 73 (CDC73), also known as parafibromin or hyperparathyroidism 2, locates primarily in the nucleus of cells and regulates the transcription of genes involved in cell growth and division [15–17]. CDC73 can also be found outside the nucleus [18, 19]. Ectopic CDC73 expression leads to the downregulation of ER stress-related proteins, suggesting its potential role in ER stress regulation [20]. ER stress has been confirmed to play a vital role in hepatic I/R injury [21, 22]. Physical interactions between HSF2BP and CDC73 were detected using yeast two-hybrid screening [23]. However, the HSF2BP-CDC73 interaction in liver disease and its regulatory effects on ER stress are still unknown.

We hypothesized that HSF2BP plays an indispensable role in the regulation of ER stress. Our objectives were as follows: (1) to measure HSF2BP expression in tunicamycin-induced ER stress; (2) to evaluate the role of HSF2BP in hepatocyte-specific HSF2BP transgenic and knockout mice during ER stress; and (3) to verify the effect of HSF2BP on ER stress and explore its underlying mechanism in hepatic I/R injury.

## RESULTS

### HSF2BP upregulation during tunicamycin-induced ER stress

To determine the role of HSF2BP in ER stress, we detected HSF2BP expression in tunicamycin (an ER stress inducer)-treated mice and HL-7702 cells. Liver damage (Additional file 1: Fig. S1A, B, p < 0.05) and ER stress (Additional file 1: Fig. S1C, D, p < 0.05) were induced by tunicamycin in mice. HSF2BP expression increased at 3 h and started to decrease at 6 h following tunicamycin treatment in mice (Additional file 1: Fig. S1C and E, p < 0.05). In HL-7702 cells, GRP78 expression started to increase at 6 h and peaked at 12 h post-treatment (Additional file 1: Fig. S2A, B, p < 0.05); in contrast, HSF2BP expression increased at 3 h and started to decrease at 6 h post-treatment (Additional file 1: Fig. S2A and C, p < 0.05).

### HSF2BP overexpression and knockout mice

To clarify the function of HSF2BP in ER stress, we constructed hepatocellular-specific overexpression and knockout mice. HSF2BP overexpression mice were produced by specifically knockin hsf2bp at the locus of ROSA26 in C57BL/6 mice, which resulted in liver-specific HSF2BP mRNA and protein expression (Additional file 1: Fig. S3A–E, p < 0.05). The TG mice displayed normal body weight and liver histology (Additional file 1: Fig. S3F–G). Hepatocyte-specific HSF2BP overexpression did not affect the mRNA expression of HSF1, HSF2, and heat shock proteins, including HSPa1a, HSPa1b, HSP90aa1, and HSP90ab1 (Additional file 1: Fig. S3H) or the mRNA expression of inflammatory factors (Additional file 1: Fig. S3I).

Hepatocyte-specific HSF2BP knockout mice were produced by deleting exon 5 of the HSF2BP gene. Hepatocyte-specific HSF2BP knockout was verified by quantitative PCR, western blot analysis, and immunohistochemical staining (Additional file 1: Fig. S4A–E, p < 0.05). Hepatocyte-specific HSF2BP knockout had no detectable effects on body weight, liver histology, the mRNA expression of HSFs and HSPs, or the mRNA expression of inflammatory factors (Additional file 1: Fig. S4F–I).

### HSF2BP mitigated tunicamycin-induced ER stress

**Fig. 1:**
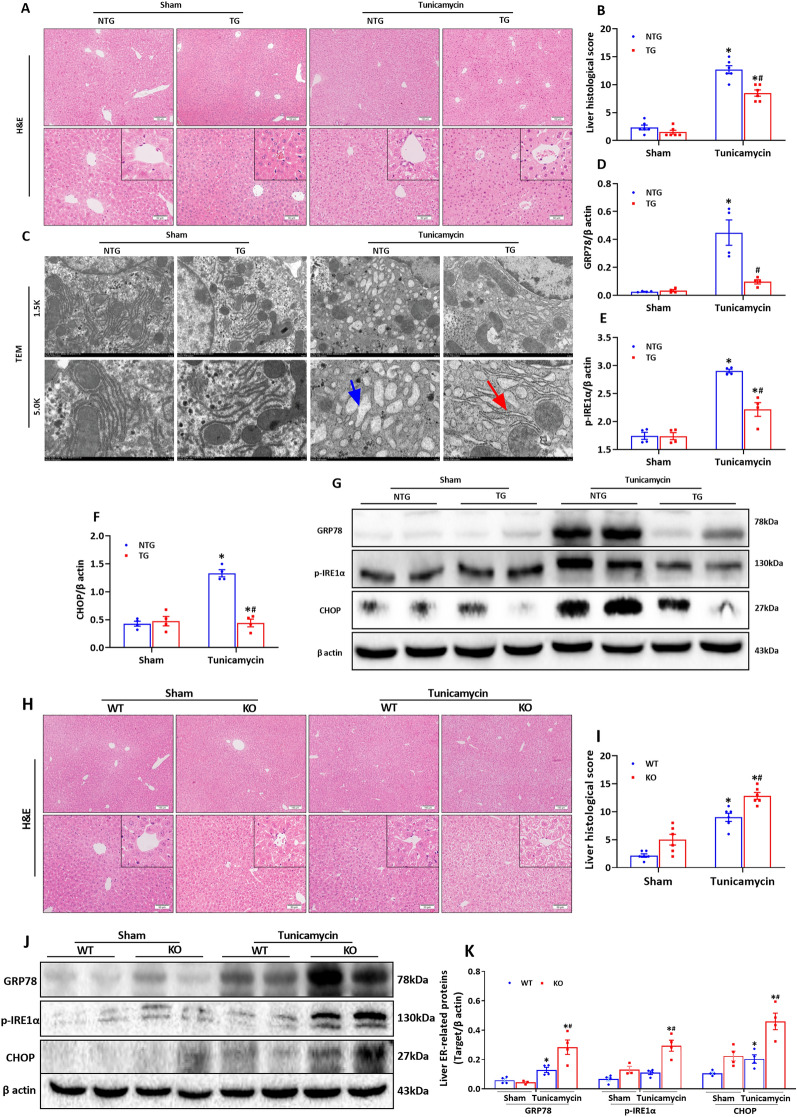
HSF2BP mitigates tunicamycin-induced ER stress. ER stress was induced by intraperitoneal injection of tunicamycin (0.5 µg/g body weight, 500 µl) for 48 h in mice. Sham mice treated with 500 µl normal saline. Liver H&E staining (**A**) and its histological score (**B**) in tunicamycin-induced ER stress in HSF2BP-TG and NTG mice. Original magnification, x100 and x200. **C**, Transmission electron microscope (TEM) of endoplasmic reticulum (ER) in tunicamycin-induced ER stress in HSF2BP-TG and NTG mice. Original magnification, x1.5k and x5.0k. Western blot analysis of ER stress-related proteins (**G**) and their quantitative results of GRP78 (**D**), p-IRE1α (**E**) and CHOP (**F**) in tunicamycin-induced ER stress in HSF2BP-TG and NTG mice. Results are expressed as mean ± SE (n = 4–6/group) and compared by one-way ANOVA. * p < 0.05 versus sham mice, ^#^ p < 0.05 versus NTG mice. The liver H&E staining (**H**) and its histological score (**I**) in tunicamycin-induced ER stress in HSF2BP-KO and WT mice. Original magnification, x100 and x200. Western blot analysis of GRP78, p-IRE1α and CHOP (**J**) and their quantitative results (**K**) in tunicamycin-induced ER stress in HSF2BP-KO and WT mice. Results are expressed as mean ± SE (n = 4–6/group) and compared by one-way ANOVA. * p < 0.05 versus sham mice, ^#^ p < 0.05 versus WT mice

In HSF2BP-transgenic (TG) mice, tunicamycin treatment led to milder liver injury than that in the non-transgenic (NTG) controls (Fig. 1A, B, p < 0.05). The HSF2BP-transgenic mice had reduced post-tunicamycin treatment dissolution of hepatic endoplasmic reticulum compared with the non-transgenic controls (Fig. 1C, p < 0.05). The HSF2BP-transgenic mice had reduced hepatic expression of ER stress-related proteins (e.g., GRP78, p-IRE1α, and CHOP) following tunicamycin treatment compared with the non-transgenic controls (Fig. 1D–G, p < 0.05). However, liver injury (Fig. 1H, I, p < 0.05) and ER stress (Fig. 1J and K, p < 0.05) were aggravated in the HSF2BP-knockout (KO) mice compared with the wild-type (WT) controls.

### HSF2BP upregulation during hepatic I/R injury in patients and mice

A hepatic I/R injury model was established to further verify the regulatory effect of HSF2BP on ER stress. First, the expression of HSF2BP in hepatic I/R injury was detected. In human liver transplant and control samples, the post-I/R liver graft had higher HSF2BP levels than the control liver without I/R injury (Additional file 1: Fig. S5A**–**D, p < 0.05). In the mice subjected to hepatic I/R, HSF2BP expression corresponded with the reperfusion time; it peaked at 2 h (Additional file 1: Fig. S5E, F, p < 0.05). In cultured hepatocytes, HSF2BP levels increased following H/R treatment, with HSF2BP expression showing a trend similar to that in the mouse hepatic I/R model (Additional file 1: Fig. S5G, H, p < 0.05).

### HSF2BP overexpression and minimized hepatic I/R injury

**Fig. 2:**
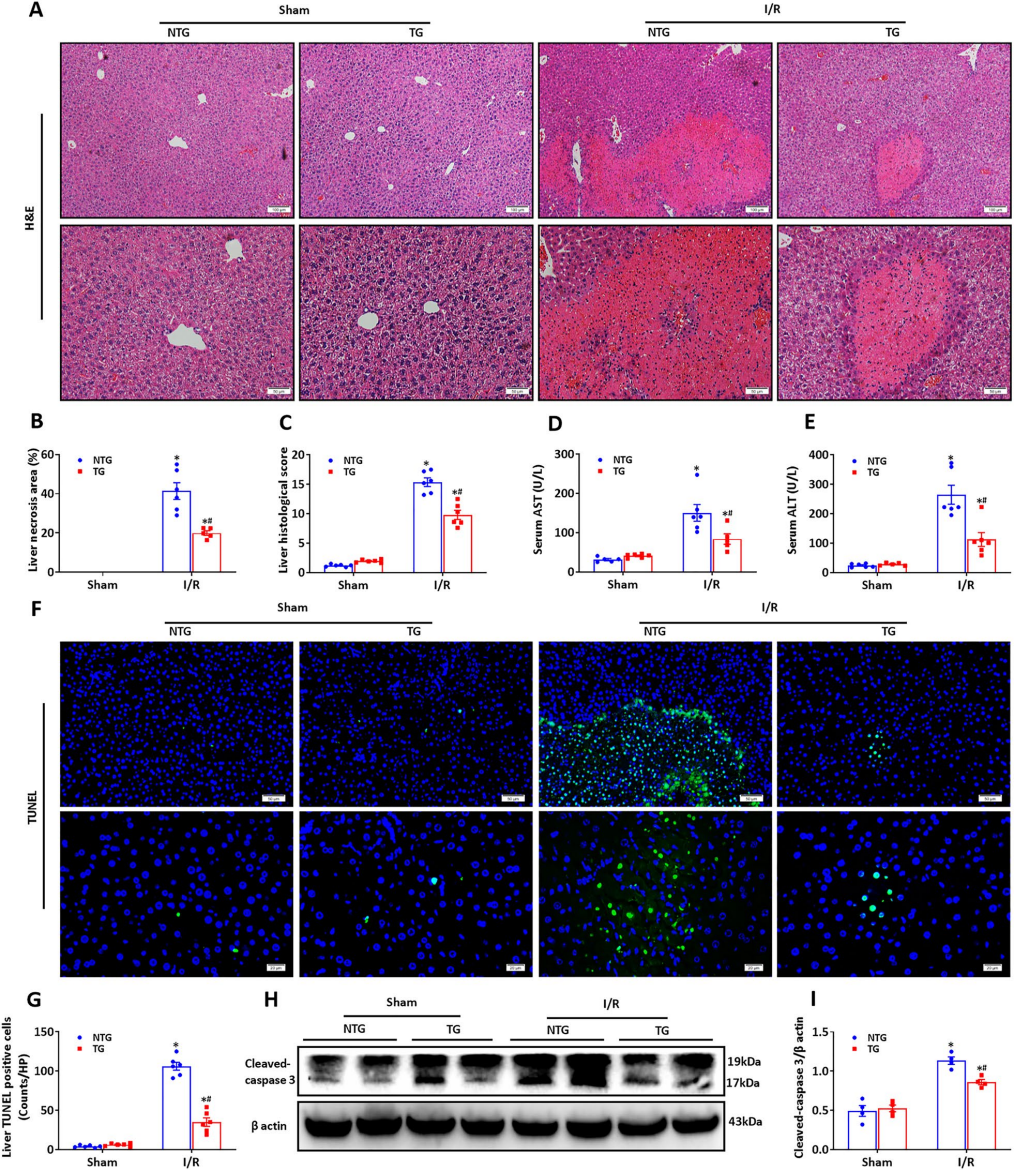
HSF2BP overexpression protects against hepatic I/R injury. Liver is induced by 1-hour ischemia followed by 24-hour reperfusion in HSF2BP-TG and NTG mice. Sham mice received all procedures except for hepatic ischemia. **A**, Liver H&E staining after hepatic I/R in HSF2BP-TG and NTG mice. Original magnification, x100 and x200. Liver necrosis area (**B**) and histological score (**C**) after hepatic I/R in HSF2BP-TG and NTG mice. Serum AST (**D**) and ALT (**E**) after hepatic I/R in HSF2BP-TG and NTG mice. Liver TUNEL staining (**F**) and its quantitative result (**G**) after hepatic I/R in HSF2BP-TG and NTG mice. Original magnification, x200 and x400. Western blot analysis of cleaved-caspase 3 (**H**) and its quantitative result (**I**) after hepatic I/R in HSF2BP-TG and NTG mice. Results are expressed as mean ± SE (n = 4–6/group) and compared by one-way ANOVA. * p < 0.05 versus sham mice, ^#^ p < 0.05 versus NTG mice

Then, transgenic mice with hepatocyte-specific HSF2BP overexpression and their non-transgenic counterparts were exposed to hepatic I/R injury. The non-transgenic mice showed characteristic I/R-induced liver injury based on H&E staining (Fig. 2A). In contrast, the transgenic mice had milder I/R-induced liver injury (Fig. 2A). The liver necrosis area and histological score were lower in transgenic mice than in non-transgenic mice after hepatic I/R injury based on quantitative analysis (Fig. 2B, C, p < 0.05). HSF2BP overexpression was associated with reduced serum AST and ALT in the transgenic mice compared with the non-transgenic mice after 24 h of reperfusion (Fig. 2D, E, p < 0.05). HSF2BP overexpression was associated with reduced TUNEL positive cells’ counts compared with the controls (Fig. 2F, G, p < 0.05). The transgenic mice had lower cleaved-caspase 3 levels than the non-transgenic mice after hepatic I/R (Fig. 2H, I, p < 0.05).

### HSF2BP overexpression and reduced inflammatory response during hepatic I/R injury

**Fig. 3:**
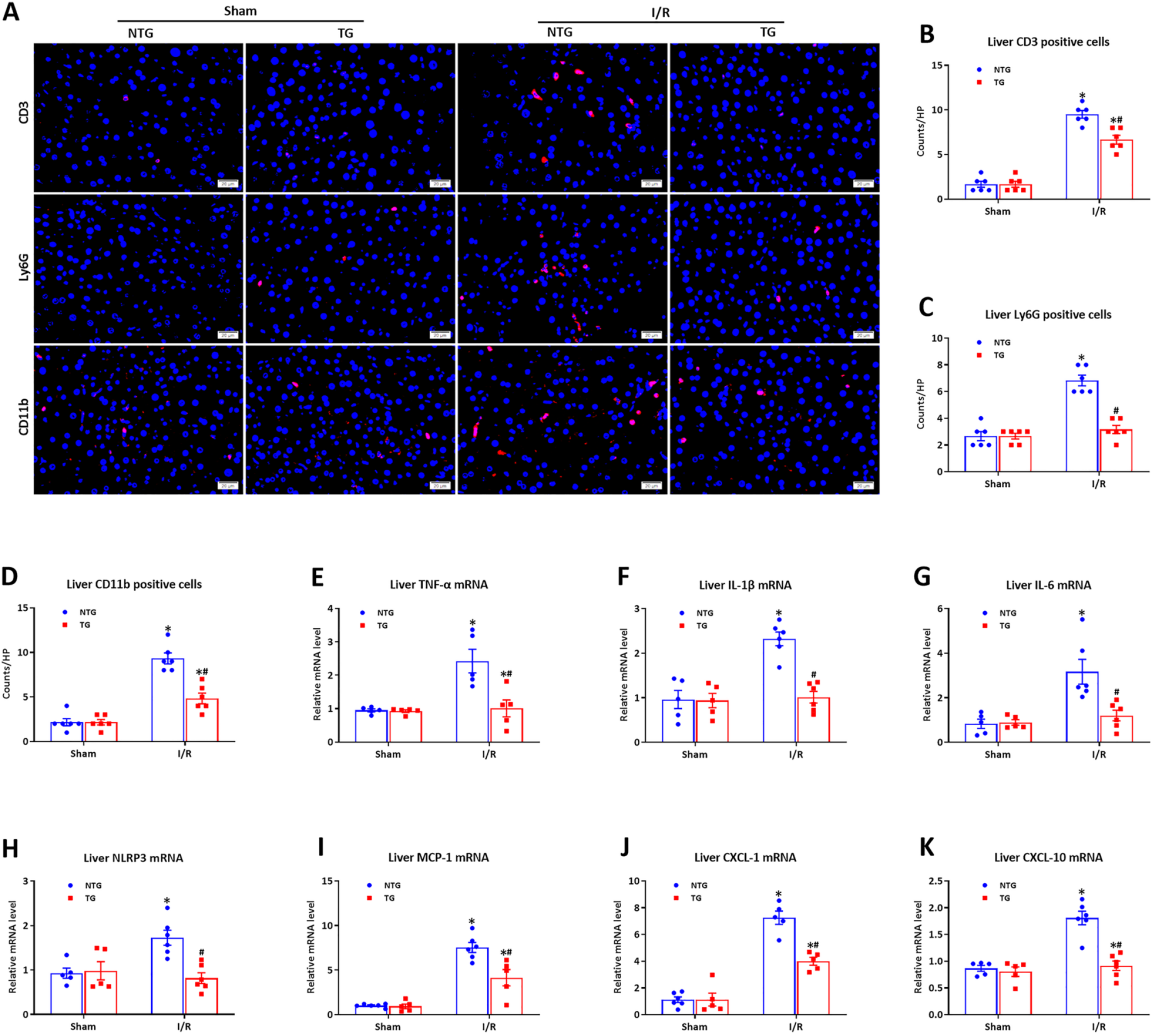
HSF2BP overexpression inhibits inflammatory response induced by hepatic I/R. Liver is induced by 1-hour ischemia followed by 24-hour reperfusion in HSF2BP-TG and NTG mice. Sham mice received all procedures except for hepatic ischemia. Liver CD3, Ly6G and CD11b staining (**A**) and their quantitative results (**B–D**) after hepatic I/R in HSF2BP-TG and NTG mice. Original magnification, x400. The mRNA levels of liver TNF-α (**E**), IL-1β (**F**), IL-6**(G)**, NLRP3 (**H**), MCP-1 (**I**), CXCL-1 (**J**), CXCL-10 (**K**) after hepatic I/R in HSF2BP-TG and NTG mice. Results are expressed as mean ± SE (n = 4–6/group) and compared by one-way ANOVA. * p < 0.05 versus sham mice, ^#^ p < 0.05 versus NTG mice

Inflammation is crucial for I/R-induced liver injury [24]. Liver CD3, Ly6G and CD11b levels were assessed by immunofluorescent staining. As revealed in Fig. 3A–D, HSF2BP overexpression was associated with reduced inflammatory cell infiltration (CD3, Ly6G, and CD11b immunofluorescent staining) compared with the controls after hepatic I/R (p < 0.05). The HSF2BP-transgenic mice had lower inflammatory factors in the mRNA level (TNF-α, IL-1β, IL-6, NLRP3, MCP-1, CXC-1, and CXCL10 based on quantitative PCR) than the controls after hepatic I/R (Fig. 3E–K, p < 0.05).

### HSF2BP overexpression and reduced ER stress during hepatic I/R injury

**Fig. 4:**
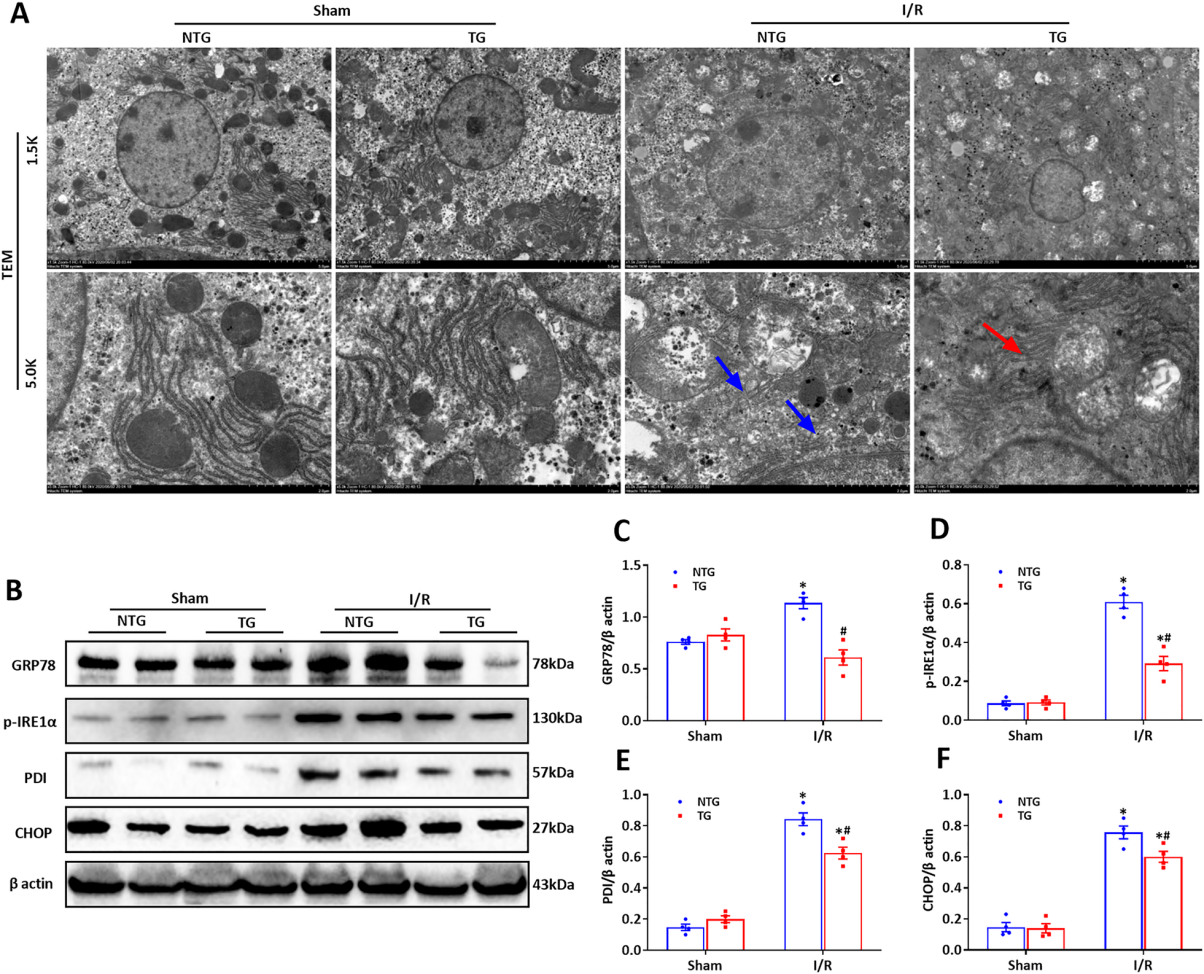
HSF2BP overexpression reduces ER stress induced by hepatic I/R. Liver is induced by 1-hour ischemia followed by 24-hour reperfusion in HSF2BP-TG and NTG mice. Sham mice received all procedures except for hepatic ischemia. **A**, Transmission electron microscope of liver endoplasmic reticulum (ER) after hepatic I/R in HSF2BP-TG and NTG mice. Original magnification, x1.5k and x5.0k. Western blot analysis of ER stress-related proteins (**B**) and their quantitative results of GRP78 (**C**), p-IRE1α (**D**), PDI (**E**) and CHOP (**F**) after hepatic I/R in HSF2BP-TG and NTG mice. Results are expressed as mean ± SE (n = 4–6/group) and compared by one-way ANOVA. * p < 0.05 versus sham mice, ^#^ p < 0.05 versus NTG mice

Growing evidence suggests that ER stress is an important pathogenic mechanism in hepatic I/R injury [25]. ER stress, characterized by ER fragmentation and disintegration observed using a transmission electron microscope, was significantly mitigated in the HSF2BP overexpressing mice compared with the controls (Fig. 4A, p < 0.05). The HSF2BP overexpressing mice also had reduced expression of proteins related to ER stress (GRP78, p-IRE1α, PDI, and CHOP) (Fig. 4B–F, p < 0.05).

### HSF2BP deficiency and exacerbated I/R-induced liver injury

**Fig. 5:**
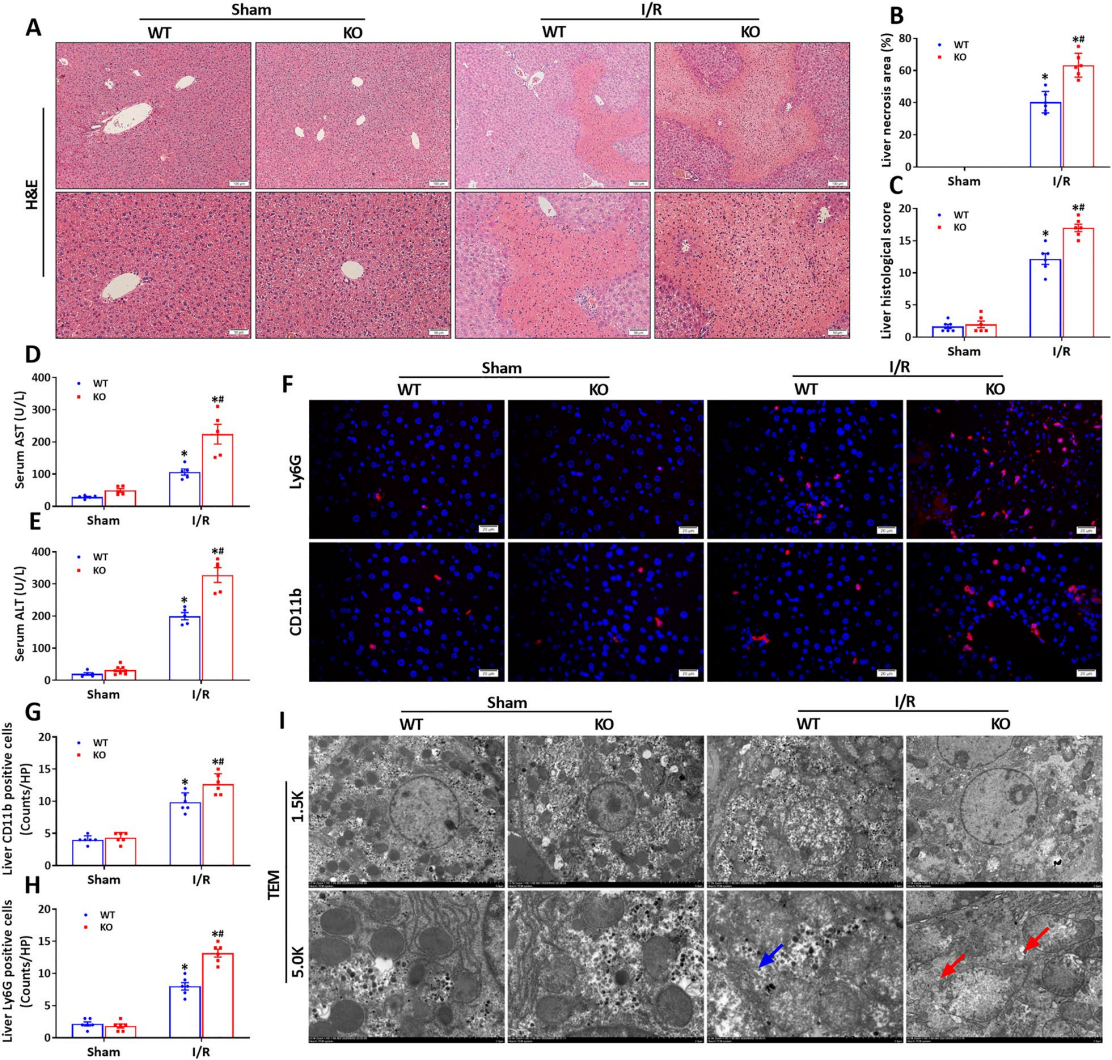
HSF2BP deficiency exacerbates I/R-induced liver injury, inflammatory response and ER stress. Liver is induced by 1-hour ischemia followed by 24-hour reperfusion in HSF2BP-KO and WT mice. Sham mice received all procedures except for hepatic ischemia. ER stress was induced by intraperitoneal injection of tunicamycin (0.5 µg/g body weight, 500 µl) for 48 h in HSF2BP-TG and NTG mice. Sham mice treated with 500 µl normal saline. **A**, Liver H&E staining after hepatic I/R in HSF2BP-KO and WT mice. Original magnification, x100 and x200. Liver necrosis area (**B**) and histological score (**C**) after hepatic I/R in HSF2BP-KO and WT mice. Serum AST **(D)** and ALT (**E**) after hepatic I/R in HSF2BP-KO and WT mice. Liver CD3 and Ly6G staining (**H**) and their quantitative results (**F** and **G**) after hepatic I/R in HSF2BP-KO and WT mice. Original magnification, x400. **I**, Transmission electron microscope of liver endoplasmic reticulum (ER) after hepatic I/R in HSF2BP-KO and WT mice. Original magnification, x1.5k and x5.0k. Results are expressed as mean ± SE (n = 4–6/group) and compared by one-way ANOVA. * p < 0.05 versus sham mice, # p < 0.05 versus NTG mice

HSF2BP knockout mice were exposed to hepatic I/R injury. The HSF2BP-deficient mice had exaggerated I/R-induced liver injury (Fig. 5A–E, p < 0.05), inflammation (Fig. 5F–H, p < 0.05) and ER stress (Fig. 5I, p < 0.05) compared with the WT controls.

### HSF2BP overexpression and increased cytoplasmic CDC73 levels

**Fig. 6:**
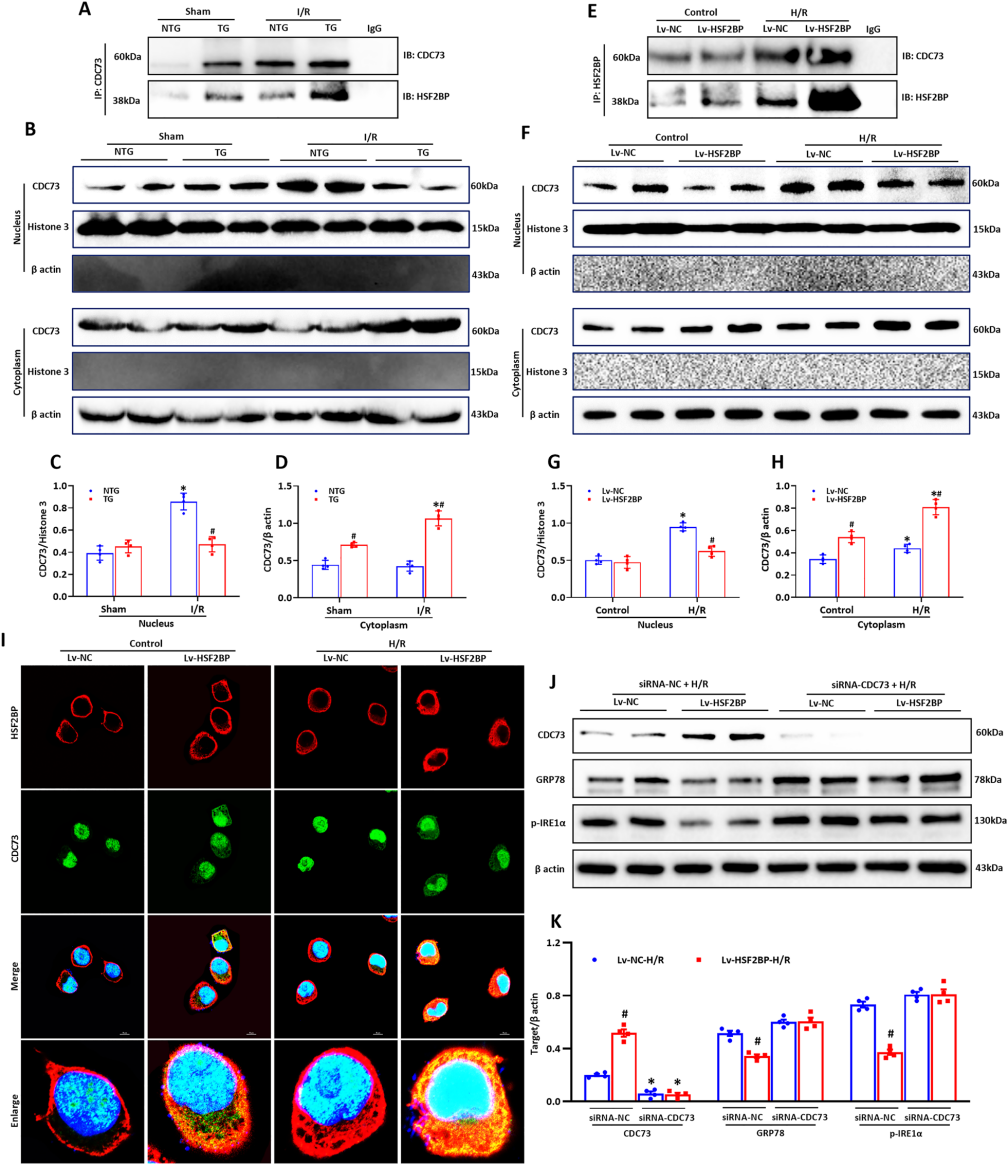
HSF2BP interacts with CDC73 and regulates its subcellular localization. Liver is induced by 1-hour ischemia followed by 24-hour reperfusion in HSF2BP-KO and WT mice. Sham mice received all procedures except for hepatic ischemia. HL-7702 cells were transfected with HSF2BP overexpressed lentivirus (Lv-HSF2BP) or negative control lentivirus (Lv-NC) and treated under hypoxia/reoxygenation (H/R) condition (Culture under glucose/FBS free 1640 medium and hypoxia condition for 1 h, then transferred to glucose/FBS-rich medium and normoxia condition for 8 h). **A**, Western blot analysis of HSF2BP and CDC73 after coimmunoprecipitation in mice. Western blot analysis of CDC73 in the nucleus and cytoplasm (**B**) and their quantitative results (**C, D**) in mice. **E**, Western blot analysis of HSF2BP and CDC73 after coimmunoprecipitation in HL-7702 cells. Western blot analysis of CDC73 in the nucleus and cytoplasm (**F**) and their quantitative results (**G, H**) in HL-7702 cells. **I**, Immunofluorescent staining of HSF2BP and CDC73 in HL-7702 cells. Results are expressed as mean ± SE (n = 3–4/group) and compared by one-way ANOVA. * p < 0.05 versus control group, ^#^ p < 0.05 versus Lv-NC group. Western blot analysis of CDC73, GRP78 and p-IRE1α (**J**) and their quantitative results (**K**) in HL-7702 cells transfected with siRNA-CDC73. Results are expressed as mean ± SE (n = 3–4/group) and compared by one-way ANOVA. * p < 0.05 versus siRNA-NC group, ^#^ p < 0.05 versus Lv-NC-H/R group

HSF2BP has been shown to interact with CDC73 [23]. As shown in Additional file 1: Fig. S6A–D, CDC73 levels were obviously induced in liver tissues from both patients and mice subjected to I/R injury (p < 0.05). Hepatocyte HSF2BP overexpression further increased CDC73 expression after hepatic I/R (Additional file 1: Fig. S6C, D, p < 0.05). HSF2BP-overexpressing mice and HL-7702 cells transfected with lentivirus for HSF2BP overexpression (Lv-HSF2BP, Additional file 1: Fig. S7A–C) were used to explore the potential role of CDC73 in HSF2BP suppression of ER stress. HSF2BP and CDC73 were physically bound in the HSF2BP-overexpressing mice based on immunoprecipitation experiments (Fig. 6A). HSF2BP overexpression increased cytoplasmic CDC73 levels, which were further increased after hepatic I/R (Fig. 6B–D). These results were confirmed in HL-7702 cells (Fig. 6E–H) Furthermore, immunofluorescence staining showed that CDC73 was primarily located in the nucleus of HL-7702 cells transfected with Lv-HSF2BP or negative control lentivirus (Lv-NC) (Fig. 6I). H/R treatment increased cytoplasmic CDC73 levels in the HSF2BP-overexpressing cells, whereas, in the Lv-NC-transfected cells, H/R treatment increased nuclear CDC73 expression, and the increased cytoplasmic CDC73 colocalized with HSF2BP in the cytoplasm (Fig. 6I). These findings were in accordance with the results of western blot analysis of cytoplasmic and nuclear CDC73 levels.

### The role of CDC73 in HSF2BP-associated inhibition of ER stress

CDC73 was knocked down using siRNA in HL-7702 cells transfected with Lv-HSF2BP or Lv-NC to investigate the role of CDC73 in the HSF2BP-associated inhibition of ER stress. The effect of HSF2BP overexpression on ER stress (GRP78 and p-IRE1α) following H/R treatment was lost following CDC73 knockdown (Fig. 6J, K).

### HSF2BP overexpression and inhibition of the Jun N-terminal kinase (JNK) signaling pathway

**Fig. 7:**
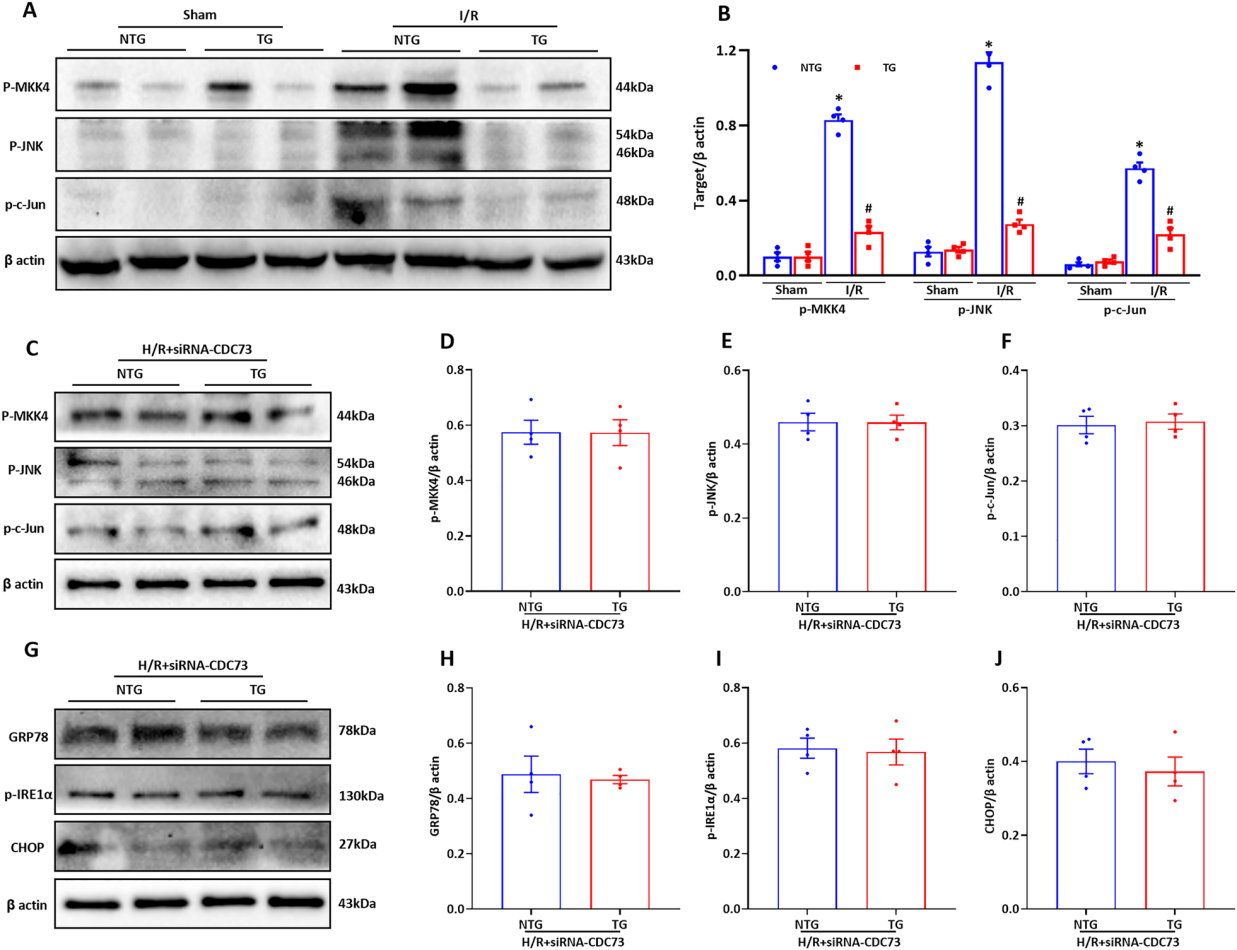
HSF2BP inhibits JNK signaling via a CDC73-dependent pathway. Liver is induced by 1-hour ischemia followed by 24-hour reperfusion in HSF2BP-TG and NTG mice. Sham mice received all procedures except for hepatic ischemia. Primary hepatocytes of HSF2BP-TG and NTG mice were isolated and treated under hypoxia/reoxygenation (H/R) condition (Culture under glucose/FBS free 1640 medium and hypoxia condition for 1 h, then transferred to glucose/FBS-rich medium and normoxia condition for 8 h). Western blot analysis of JNK signaling (p-MKK4, p-JNK and p-c-Jun) (**A**) and their quantitative results (**B**) after hepatic I/R in HSF2BP-TG and NTG mice. Results are expressed as mean ± SE (n = 4–6/group) and compared by one-way ANOVA. * p < 0.05 versus sham mice, ^#^ p < 0.05 versus NTG mice. Western blot analysis of JNK signaling (p-MKK4, p-JNK and p-c-Jun) (**C**) and their quantitative results (**D–F**) in primary hepatocytes isolated from HSF2BP-TG and NTG mice and transfected with siRNA-CDC73. Western blot analysis of ER stress-related proteins (GRP78, p-IRE1α and CHOP) (**G**) and their quantitative results (**H–J**) in primary hepatocytes isolated from HSF2BP-TG and NTG mice and transfected with siRNA-CDC73. Results are expressed as mean ± SE (n = 3–4/group) and compared by t-test. * p < 0.05

The JNK signaling pathway plays a vital role in I/R injury of liver [26]. The JNK signaling pathway (p-MKK4, p-JNK, and p-c-Jun) was detected using western blot analysis to investigate the JNK signaling pathway’s role in the HSF2BP-exerted effects. Hepatic I/R elevated expression of p-MKK4, p-JNK, and p-c-Jun, suggesting activated JNK signaling (Fig. 7A, B, p < 0.05). Hepatocyte-specific HSF2BP overexpressing mice had significantly lower p-MKK4, p-JNK, and p-c-Jun levels after hepatic I/R than their non-transgenic controls (Fig. 7A, B, p < 0.05).

### The role of CDC73 in HSF2BP-associated JNK signaling inhibition

Primary hepatocytes from HSF2BP transgenic and non-transgenic mice were isolated to investigate the role of CDC73 in the HSF2BP-associated inhibition of JNK signaling (Additional file 1: Fig. S8A, B, p < 0.05). CDC73 knockdown using siRNA eliminated the inhibitory effect of HSF2BP on JNK signaling following H/R treatment (Fig. 7C–F). CDC73 RNAi also eliminated the inhibitory effect of HSF2BP on H/R-induced ER stress (Fig. 7G–J).

### The role of HSF2BP in hepatic ischemic preconditioning (HIPC)

The role of HSF2BP in hepatic ischemic preconditioning (HIPC) during hepatic I/R was evaluated. HIPC was induced by liver ischemia for 10 min, followed by 10 min of reperfusion. HIPC upregulated the expression of HSF2BP (Additional file 1: Fig. S9A, B, p < 0.05). Liver H&E staining showed that HSF2BP knockout eliminated the hepatoprotective effect of HIPC during hepatic I/R (Additional file 1: Fig. S9C–E).

## DISCUSSION

Our study, aiming to determine the regulatory role of HSF2BP on ER stress, had several important findings. We showed that (1) hepatic HSF2BP expression is increased in tunicamycin-induced ER stress, (2) HSF2BP is hepatoprotective in tunicamycin-induced ER stress, as evidenced through extensive gain-of-function and loss-of-function experiments, and (3) HSF2BP protects against I/R-induced liver injury, inflammation, ER stress and subsequent activation of the JNK signaling pathway, likely via CDC73 subcellular localization regulation. These results indicate that HSF2BP is a previously uncharacterized regulatory factor of ER stress and may be a novel target for the prevention and treatment of ER stress-related liver diseases.

As mentioned above, HSF2BP has a variety of physiological functions and plays an important role in a variety of diseases. Our recent study found that HSF2BP protects against acute liver injury by regulating HSF2/HSP70/MAPK signaling [14]. However, multiple studies have found that HSF2BP can also function independently of HSF2 [4, 5, 27]. In this regard, the role of HSF2BP in other liver diseases, and whether there is a mechanism independent of HSF2, warrant investigation.

We generated transgenic mice with hepatocyte-specific HSF2BP overexpression and knockout mice with hepatocyte-specific HSF2BP deletion to directly evaluate the biological importance of HSF2BP in hepatic I/R injury. The transgenic mice showed robust HSF2BP mRNA expression in the liver. The HSF2BP protein levels in transgenic mice were also significantly increased. The knockout mice, in contrast, had no HSF2BP expression in hepatocytes. Neither the transgenic mice nor the knockout mice displayed detectable notable phenotypes. HSF2BP overexpression or knockout did not result in meaningful changes in the mRNA expression of HSF1, HSF2, and heat shock proteins, suggesting that HSF2BP plays a minor role in regulating heat shock responses under non-stress conditions. This finding is consistent with the fact that, although HSF2BP was discovered as a binding protein of HSF2, none of its reported functions are related to the regulation of heat shock responses [6–35].

The ER regulates protein synthesis, folding, and trafficking [25]. Hepatocytes are prone to ER stress-related injury due to their abundant ER content. ER homeostasis perturbation can lead to the generation of unfolded proteins under pathological conditions [28]. ER stress ensues if the signal transduction cascade triggering called the unfolded protein reaction, which is tasked to restore protein homeostasis, is unsuccessful [29]. Our present study found that the expression of HSF2BP was increased during ER stress. Liver injury, inflammatory response and ER stress were reduced in hepatocyte-specific HSF2BP overexpressing mice after treatment with tunicamycin, but were aggravated in hepatocyte-specific HSF2BP knockout mice, suggesting that HSF2BP was a potential regulator of ER stress. ER stress plays an indispensable role in hepatic I/R injury [30], and is closely related to the inflammatory response [31]. Inhibition of ER stress is thought to have a hepatoprotective during hepatic I/R injury [32]. The regulatory effect of HSF2BP on ER stress was further confirmed in hepatic I/R injury model. Together, these results suggest that HSF2BP is a vital moderator of ER stress in the liver.

ER stress induces the activation of the JNK signaling pathway [26], which leads to multiple biological events, including increased inflammation and hepatocyte death [33–35]. In addition, a study found that HSF2BP could promote cell proliferation by regulating the JNK signaling pathway [13]. Here, we found that HSF2BP-overexpressing mice had lower-levels of phosphorylated MKK4, JNK, and c-Jun after hepatic I/R injury that the NTG controls, suggesting attenuated JNK signaling. The inhibition of JNK signaling by HSF2BP may be related to its regulation of ER stress. Overactivation of JNK signaling can induce liver injury under various conditions [36], and attenuation of the JNK signaling has been shown to inhibit hepatic injury in liver diseases [37]. Thus, the decreased activity of the JNK signaling pathway is likely responsible, at least in part, for the protective effect exerted by HSF2BP in hepatic I/R injury.

In terms of the possible molecular mechanism, we found that HSF2BP directly binds to CDC73, and CDC73 knockdown eliminated the inhibitory effect of HSF2BP on ER stress and JNK signaling in hepatocytes, suggesting the critical role of the HSF2BP/CDC73 interaction in the hepatocellular protective effect of HSF2BP. Importantly, our results revealed that HSF2BP overexpression likely sequesters CDC73 in hepatocyte cytoplasmic compartments during H/R treatment. CDC73 is considered a predominantly nuclear protein that regulates gene transcription through interaction with RNA polymerase II [38]; however, a small fraction of CDC73 can be translocated to the cytoplasm, a process that might be actively regulated [18, 19]. Cytoplasmic CDC73 may possess various biological functions. Zheng et al. revealed that cytoplasmic CDC73 enhances proliferation and suppresses apoptosis in colorectal cancer cells [39]. Jo et al. found that CDC73 regulates p53-mediated apoptosis by directly targeting and destabilizing p53 mRNA in the cytoplasm [19]. Zhao et al. showed the downregulation of ER stress-related proteins, including GRP78 via plasmid-mediated ectopic expression of CDC73 in DLD-1 colon carcinoma cells [20], which appears to be consistent with our findings. Our results showed that HSF2BP was mainly expressed in the cytoplasm and CDC73 was mainly expressed in the nucleus. We believe some CDC73 migrated from the nucleus to the cytoplasm during hepatic I/R injury and high levels of cytoplasmic HSF2BP sequestered CDC73 to the cytoplasm. This finding is very similar to another study which showed that HSF2BP represses basonuclin (BNC1) transcriptional activity by sequestering BNC1 to the cytoplasm in HEK293T cells [4]. However, whether HSF2BP/CDC73 interaction also occurs in the nucleus and the detailed molecular events induced by cytoplasmic CDC73 still needs to be further explored. In addition, HSF2BP knockout mice showed some signs of liver injury and ER stress in tunicamycin-induced ER stress. This finding may be related to the slight decrease of CDC73 in the cytoplasm after HSF2BP knockout, suggesting the possible role of HSF2BP in maintaining normal liver function and more studies are still needed.

Hepatic ischemic preconditioning (HIPC) is an effective surgical approach to alleviate hepatic I/R. However, the exact mechanism is still not well defined [40, 41]. In our study, HIPC upregulated the expression of HSF2BP, and HSF2BP knockout eliminated the hepatoprotective effect of HIPC during hepatic I/R, which suggested that HSF2BP may be a crucial factor in HIPC during hepatic I/R. However, the specific mechanism still needs further verification.

In summary, our study demonstrates that HSF2BP is a potential regulator of ER stress, likely via the regulation of CDC73 subcellular localization. HSF2BP is a promising hepatoprotective factor and deserves further research for its potential prophylactic and therapeutic effects in a variety of ER stress-related liver diseases.

## Materials and methods

### Human liver samples

Human liver samples of healthy controls and liver transplants patients were obtained at the First Affiliated Hospital of Xi’an Jiaotong University, China. Donor liver tissues during liver transplantation were obtained shortly after blood flow restoration and used as hepatic I/R samples (n = 4, 3 cases of brain trauma, 1 case of severe cerebral infarction). Liver tissues from patients with cholecystectomy without hepatitis, steatosis, and I/R injuries were used as controls (n = 3). All patients in the two groups were male and age matched. All subjects signed the informed consent forms and were approved by the Internal Review Board of the First Affiliated Hospital of Xi’an Jiaotong University. Patients were not involved in the design, conduct, reporting or dissemination plans of the study.

### Generation of transgenic mice with hepatocyte-specific HSF2BP overexpression

Hepatocyte-specific HSF2BP-overexpressing mice were generated at Cyagen Bioscience (Suzhou, China) and used by us previously [14]. In brief, mouse hsf2bp knockin was created at the locus of ROSA26 in C57BL/6J mice using CRISPR/Cas9 knock-in technique. The “Alb-mouse hsf2bp CDS-polyA” cassette was reversely inserted into intron 1 of ROSA26. The BAC clone of C57BL/6J library was used as a template for donor vector engineering to generate homologous arms by PCR. A donor vector carrying Cas9 and gRNA was injected into C57BL/6J zygotes. Tail-derived DNA from the pups was used for genotyping. HSF2BP overexpression was confirmed by quantitative PCR analysis of HSF2BP mRNA in various organs (Additional file 1: Fig. S3).

### Generation of knockout mice with hepatocyte-specific HSF2BP knockout

Hepatocyte-specific HSF2BP knockout (KO) mice were generated at Cyagen Bioscience (Suzhou, China) using the CRISPR-Cas 9 technique and used by us previously [14]. Briefly, exon 5 of the hsf2bp gene was selected as the conditional knockout region (cKO region). Using a BAC clone from the C57BL/6J library as a template, the homologous arm and cKO region were obtained by PCR to construct the target vector. The Neo cassette had self-deletion anchor sites on targeting vector. DTA was chosen for negative selection and ES cells of C57BL/6J mice were chosen for gene targeting. Hsf2bp-floxed mice were hybridized with albumin-Cre mice under the C57BL/6J background to obtain hepatocyte-specific HSF2BP knockout mice. HSF2BP knockout was confirmed by quantitative PCR analysis of HSF2BP mRNA in various organs (Additional file 1: Fig. S4).

### Mouse hepatic I/R model

The mouse model of hepatic I/R injury was established as described previously [42]. Briefly, hepatic ischemia was induced by partially occluding (70%) hepatic arterial/portal venous blood using a microvascular clip. One hour later, the microvascular clip was removed, and reperfusion resumed. Sham mice underwent the same procedure except for hepatic ischemia.

### Animal care

All mice were fed standard diets under normal conditions. Male mice (aged 8 to 10 weeks and weighing 20 to 25 g) were used for the study. This project was approved by the Institutional Animal Care and Use Committee of the Ethics Committee of Xi’an Jiaotong University Health Science Center and carried out according to the China Council’s guidelines on Animal Care and Use.

### Hematoxylin & eosin staining

The liver tissues fixed in paraformaldehyde (4%) for 48 h were embedded in paraffin. Five-millimeter slices were cut for Hematoxylin & Eosin staining. An established grading system was used to evaluate the liver histological score [43]. In brief, the liver histological score was the sum of the individual scores of the following 6 items: cytoplasmic color fading, vacuolization, nuclear condensation, nuclear fragmentation, nuclear fading, and erythrocyte stasis (0, no; 1, mild; 2, moderate; and 3, severe, ranging from 0 to 18). The liver necrosis area was evaluated by Image J software.

### Aspartate aminotransferase (AST)/alanine aminotransferase (ALT) measurement

The AST assay kit (C010-2) and ALT assay kit (C009-2) were used for serum AST and ALT measurements according to instructions (Nanjing Jiancheng Bioengineering Institute, Nanjing, China).

### Western blot analysis

Western blot analysis was executed as described [44]. Information regarding the antibodies is showen in Additional file 1: Table S1. β actin was chosen as an endogenous control. The quantitative analysis of protein was carried out by Image J software.

### Quantitative PCR

Quantitative PCR was carried out as described previously [45]. The primer sequences (TaKaRa Bio Inc., China) are showen in Additional file 1: Table S2. β actin was used as an endogenous control.

### Immunohistochemistry/immunofluorescence staining

Immunohistochemistry and immunofluorescence staining of liver were executed as described [46] and quantified by Image J software. The reagents were used as follows: HSF2BP (ab126252, Abcam, USA), TUNEL assay (Roche, Switzerland), and CD11b/CD3/Ly6G antibody (Servicebio, China). The immunofluorescence staining of HL-7702 cells was observed using a confocal laser scanning microscope (Leica).

### Transmission electron microscope

The endoplasmic reticulum structure of hepatocytes was observed by a transmission electron microscope (HT7700, Japan) as previously described [47].

### Co-immunoprecipitation

Protein A/G PLUS-Agarose (sc‐2003, Santa) and HSF2BP antibody (sc-130,322, Santa) were used for immunoprecipitation in HL-7702 cells per instructions. Western blot analysis was conducted as described above. Histone 3 and β actin were used as endogenous controls for nuclear protein and cytoplasmic protein detection.

### Cell culture, hypoxia/reoxygenation, transfection of siRNA and lentivirus

HL-7702 cells were chosen and cultured with RPMI-1640 medium containing 10% fetal bovine serum and streptomycin/penicillin (100 U/ml) mixture in a 5% CO_2_ and 37°C environment. Hypoxia/reoxygenation (H/R) was performed as described previously [47]. siRNA (GenePharma Corporation, Shanghai, China) was transfected as directed. siRNA-CDC73 (5′ - GCU UAA GCU GGU GGA AGA AAT T ‐ 3′ and 5′ ‐ UUU CUC CAC CAG CUU AAG CTT ‐ 3′) was used to knock down the expression of CDC73, and siRNA-NC (5’ - UUC UCC GAA CGU GUC ACG UTT − 3’ and 5’ - ACG UGA CAC GUU CGG AGA ATT − 3’) was used as the negative control. The HSF2BP-overexpressing lentivirus (Lv-HSF2BP) and negative control lentivirus (Lv-NC) were synthesized by Genechem Corporation (Shanghai, China) and transfected according to the instructions.

### Statistical analysis

The results are presented as the mean ± standard error (SE). The differences between groups were examined by t-test or one-way ANOVA and analysis by SPSS software (IBM, Armonk, NY). The statistically significant threshold was 0.05.

## Supplementary information

**Additional file 1**: **Table S1. **The information of antibodies forwestern blot analysis. **Table S2. **The information of primers for q-PCR. **Figure S1.** HSF2BP expression is increased after tunicamycin-induced ER stress inmice. Liver H&E staining (A) andits histological score (B) intunicamycin-induced ER stress. Original magnification, x100 and x200. C, Western blot analysis of HSF2BP and GRP78 in tunicamycin-induced ERstress. Quantitative analysis of GRP78 (D)and HSF2BP (E) intunicamycin-induced ER stress. Results are expressed as mean ± SE (n =4-6/group) and compared by one-way ANOVA. * p < 0.05. **Figure S2.** HSF2BP expression is increased after tunicamycin-induced ERstress in cultured hepatocytes. A, Western blot analysisof HSF2BP and GRP78 in HL-7702 cells. Quantitative analysis of GRP78 (B) and HSF2BP (C) in HL-7702 cells. Results are expressed as mean ± SE (n =3/group) and compared by one-way ANOVA. * p < 0.05. **Figure S3. **Theestablishment of hepatocyte-specific HSF2BP transgenic mice. A, The HSF2BP mRNA levels in variousorgans of HSF2BP transgenic (TG) mice. Western blotanalysis of HSF2BP (B) and itsquantitative analysis (C) in liverof hepatocyte-specific HSF2BP-TG and NTG mice. The immunohistochemical stainingof HSF2BP (D) and its IHC score (E) in liver of HSF2BP-TG and NTG mice.Original magnification, x400. F, Bodyweight of HSF2BP-TG and NTG mice. G, TheH&E staining in liver of HSF2BP-TG and NTG mice. Original magnification, x400.H, The levels of HSFs (heat shock factors)and HSPs (heat shock proteins) in liver of HSF2BP-TG and NTG mice. I, The levels of inflammatory factorsin liver of HSF2BP-TG and NTG mice. Results are expressed as mean ± SE (n =4-6/group) and compared by t-test or one-way ANOVA. * p < 0.05. **Figure S4.** Theestablishment of hepatocyte-specific HSF2BP knockout mice. A, The HSF2BP mRNA levels invarious organs of HSF2BP knockout (KO) mice. Western blot analysis of HSF2BP (B) and its quantitative analysis (C) in liver of HSF2BP-KO and WT mice.The immunohistochemical staining of HSF2BP (D)and its IHC score (E) in liver ofHSF2BP-KO and WT mice. Original magnification, x400. F, Body weight of HSF2BP-KO and WT mice. G, The H&E staining in liver of HSF2BP-KO and WT mice.Original magnification, x400. H, Thelevels of HSFs (heat shock factors) and HSPs (heat shock proteins) in liver ofHSF2BP-KO and WT mice. I, The levelsof inflammatory factors in liver of HSF2BP-KO and WT mice. Results areexpressed as mean ± SE (n = 4-6/group) and compared by t-test or one-way ANOVA.* p < 0.05. **Figure S5.** HSF2BP isupregulated in hepatic I/R injury. Liver immunohistochemical staining of HSF2BP (A) and its IHCscore (B)in liver transplant patients and healthy individuals. Western blot analysis ofHSF2BP (C) and its quantitativeresult (D) in human livers of livertransplant patients (n = 4) and healthy individuals (n = 3). Western blotanalysis of HSF2BP (E) and itsquantitative result (F) in mouselivers subjected to ischemia and reperfusion (n = 4-6/group). Western blotanalysis of HSF2BP (G) and itsquantitative result (H) in culturedhepatocytes under H/R condition (n = 3/group). Results are expressed as mean ±SE and compared by t-test or one-way ANOVA. * p < 0.05 versus sham mice orcontrol group. **Figure S6.** CDC73 expressionlevels are increased in liver transplant patients and hepatic I/R mice.Western blot analysis ofCDC73 (A) and its quantitativeanalysis (B) in livers of livertransplant patients (n = 3) and healthy individuals (n = 4). Results areexpressed as mean ± SE and compared by t-test. * p < 0.05. Western blotanalysis of CDC73 (C) and itsquantitative analysis (D) in mouselivers. Results are expressed as mean ± SE (n = 4) and compared by one-wayANOVA. * p < 0.05 versus sham mice, # p < 0.05 versus NTG mice. **Figure S7. ** HSF2BP expression is increased after transfection with HSF2BPoverexpressed lentivirus in cultured hepatocytes. Western blot analysis ofHSF2BP (A) and its quantitativeanalysis (B) after transfection withLv-HSF2BP in HL-7702 cells. C, The HSF2BP mRNA level after transfection withLv-HSF2BP in HL-7702 cells. Results are expressed as mean ± SE (n = 3-5/group)and compared by t-test. * p < 0.05. **Figure S8.** CDC73 expression is knocked down after transfection with siRNA-CDC73 in primaryhepatocytes. Westernblot analysis of CDC73 (A) and itsquantitative analysis (B) aftertransfection with siRNA-CDC73 in primary hepatocytes. Results are expressed asmean ± SE (n = 4/group) and compared by one-way ANOVA. * p < 0.05 versussiRNA-NC group, # p < 0.05 versus NTG-H/R group. **Figure S9.** Hepatic ischemicpreconditioning (HIPC) up-regulated the expression of HSF2BP, and HSF2BPknockout eliminated HIPC’s hepatoprotective effect during hepatic I/R. HIPC was induced by liverischemia for 10 min, followed by 10 min of reperfusion. Western blot analysisof HSF2BP (A) and its quantitativeanalysis (B) after HIPC. Results areexpressed as mean ± SE (n = 4-6/group) and compared by t-test. * p < 0.05versus sham group. C, Liver H&Estaining. Original magnification, x100 and x200. Liver necrosis area (D) and histological score (E). Results are expressed as mean ± SE(n = 4/group) and compared by one-way ANOVA. * p < 0.05 versus WT mice, # p< 0.05 versus I/R group.

## Data Availability

All data generated or analysed during this study are included in this published article.

## Abbreviations

HSF2BP: Heat shock factor 2 binding protein
CDC73: Cell division cycle 73
ER: Endoplasmic reticulum
I/R: Ischemia–reperfusion
HSF2: Heat shock factor 2
HSPs: Heat shock proteins
HSFs: Heat shock factors
H/R: Hypoxia/reoxygenation
TG: Transgenic
NTG: Non-transgenic
KO: Knockout
WT: Wild type
q-PCR: Quantitative-PCR
H&E: Hematoxylin & Eosin
SE: Standard error
NC: Negative control
Lv: Lentivirus
siRNA: Small interfering RNA
JNK: Jun N-terminal kinase
HIPC: Hepatic ischemic preconditioning
