# Supplementary material for: Overexpression of HSF2 binding protein suppresses endoplasmic reticulum stress via regulating subcellular localization of CDC73 in hepatocytes
Source: Cell Biosci. 2023 Mar 24;13:64. doi: 10.1186/s13578-023-01010-w (PMC10039577; doi:10.1186/s13578-023-01010-w)
Supplement: Supplementary file 1 — Additional file 1: Table S1. The information of antibodies forwestern blot analysis. Table S2. The information of primers for q-PCR. Figure S1. HSF2BP expression is increased after tunicamycin-induced ER stress inmice. Liver H&E staining (A) andits histological score (B) intunicamycin-induced ER stress. Original magnification, x100 and x200. C, Western blot analysis of HSF2BP and GRP78 in tunicamycin-induced ERstress. Quantitative analysis of GRP78 (D)and HSF2BP (E) intunicamycin-induced ER stress. Results are expressed as mean ± SE (n =4-6/group) and compared by one-way ANOVA. * p < 0.05. Figure S2. HSF2BP expression is increased after tunicamycin-induced ERstress in cultured hepatocytes. A, Western blot analysisof HSF2BP and GRP78 in HL-7702 cells. Quantitative analysis of GRP78 (B) and HSF2BP (C) in HL-7702 cells. Results are expressed as mean ± SE (n =3/group) and compared by one-way ANOVA. * p < 0.05. Figure S3. Theestablishment of hepatocyte-specific HSF2BP transgenic mice. A, The HSF2BP mRNA levels in variousorgans of HSF2BP transgenic (TG) mice. Western blotanalysis of HSF2BP (B) and itsquantitative analysis (C) in liverof hepatocyte-specific HSF2BP-TG and NTG mice. The immunohistochemical stainingof HSF2BP (D) and its IHC score (E) in liver of HSF2BP-TG and NTG mice.Original magnification, x400. F, Bodyweight of HSF2BP-TG and NTG mice. G, TheH&E staining in liver of HSF2BP-TG and NTG mice. Original magnification, x400.H, The levels of HSFs (heat shock factors)and HSPs (heat shock proteins) in liver of HSF2BP-TG and NTG mice. I, The levels of inflammatory factorsin liver of HSF2BP-TG and NTG mice. Results are expressed as mean ± SE (n =4-6/group) and compared by t-test or one-way ANOVA. * p < 0.05. Figure S4. Theestablishment of hepatocyte-specific HSF2BP knockout mice. A, The HSF2BP mRNA levels invarious organs of HSF2BP knockout (KO) mice. Western blot analysis of HSF2BP (B) and its quantitative analysis (C) in liver of HSF2BP-KO and WT mice.The immunohist [file 13578_2023_1010_MOESM1_ESM.docx]

**Overexpression of HSF2 Binding Protein Suppresses Endoplasmic Reticulum Stress via Regulating Subcellular Localization of CDC73 in Hepatocytes**

Jia Zhang, Tao Wang, Jianbin Bi, Mengyun Ke, Yifan Ren, Mengzhou Wang, Zhaoqing Du, Wuming Liu, Liangshuo Hu, Xiaogang Zhang, Xuemin Liu, Bo Wang, Zheng Wu, Yi Lv, Lingzhong Meng, Rongqian Wu*

*Corresponding author: [rwu001@mail.xjtu.edu.cn](mailto:rwu001@mail.xjtu.edu.cn)

**SUPPLEMENTARY MATERIALS:**

**Table S1. The information of antibodies for western blot analysis.**

| **Antibody** | **Item No** | **Company and location** |
| --- | --- | --- |
| HSF2BP | ab126252 | Abcam, Cambridge, MA, USA |
| Cleaved caspase 3 | 9661S | Cell Signaling Technology, Beverly, MA, USA |
| GRP78 | 3183 | Cell Signaling Technology, Beverly, MA, USA |
| p-IRE1α | ab48187 | Abcam, Cambridge, MA, USA |
| PDI | 3501 | Cell Signaling Technology, Beverly, MA, USA |
| CHOP | 2895 | Cell Signaling Technology, Beverly, MA, USA |
| CDC73 | ab139187 | Abcam, Cambridge, MA, USA |
| β actin | HRP-60008 | Proteintech, China |
| Histone 3 | 17168-1-AP | Proteintech, China |
| Goat anti-rabbit IgG | SA00001-2 | Proteintech, China |
| Goat anti-mouse IgG | SA00001-1 | Proteintech, China |

**Table S2. The information of primers for q-PCR.**

| **Species** | **Gene** | **Primer sequence (5’-3’)** | |
| --- | --- | --- | --- |
|  |  | **Forward** | **Reverse** |
| Mouse | β actin | GTGACGTTGACATCCGTAAAGA | GTAACAGTCCGCCTAGAAGCAC |
| Mouse | TNF-α | GCCAGAGCCACATGCTCCTA | GATAAGGCTTGGCAACCCAAGTAA |
| Mouse | NLRP3 | CCAGACACTCATGTTGCCTGTTC | GAGGCTCCGGTTGGTGCTTA |
| Mouse | CXCL-1 | TGCACCCAAACCGAAGTC | GTCAGAAGCCAGCGTTCACC |
| Mouse | CXCL-10 | ATCCGGAATCTAAGACCATCAAGAA | TGTCCATCCATCGCAGCAC |
| Mouse | IL-1β | TCCAGGATGAGGACATGAGCAC | GAACGTCACACACCAGCAGGTTA |
| Mouse | IL-6 | CCACTTCACAAGTCGGAGGCTTA | TGCAAGTGCATCATCGTTGTTC |
| Mouse | MCP-1 | AGCAGCAGGTGTCCCAAAGA | GTGCTGAAGACCTTAGGGCAGA |
| Mouse | HSF1 | TGACAGATGTGCAGCTGATGAA | GACTGCACCAGTGAGATCAGGA |
| Mouse | HSF2 | CCGGGCTAACAATGAAGCAGA | AGCTCGCCATGTTATTGTGTTTGA |
| Mouse | HSPa1a | CAGAGGCCAGGGCTGGATTA | ACACATGCTGGTGCTGTCACTTC |
| Mouse | HSPa1b | CGCTCGAGTCCTATGCCTTCA | GGCACTTGTCCAGCACCTTC |
| Mouse | HSP90ab1 | TGCTCTGTACTACTACTCGGCTTTC | CATGAGCTGGGCAATTTCTG |
| Mouse | HSP90aa1 | CCATGCTAACAGGATCTACAGGA | TCTTCAGTTACAGCAGCACTGG |
| Mouse | HSF2BP | TTCAAAGCCCGGCTAGAAACTG | GTGCAATACTCTGCCTGCTGGA |


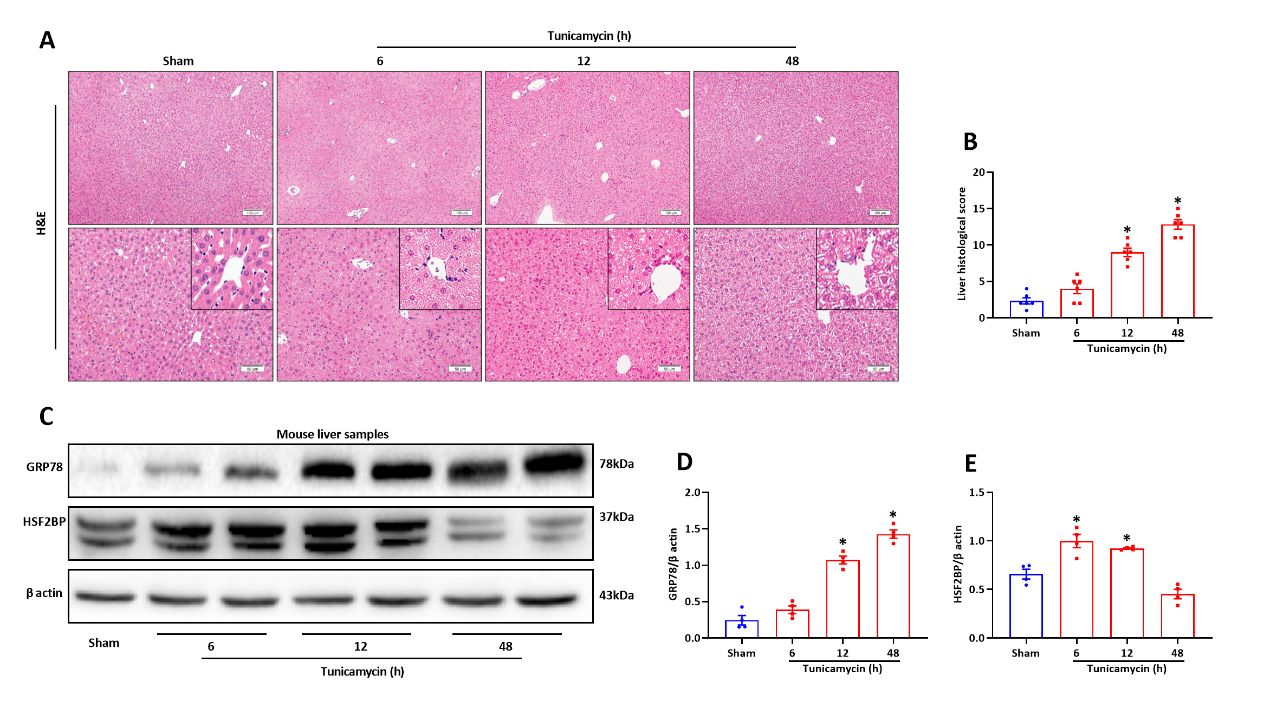


**Figure S1. HSF2BP expression is increased after tunicamycin-induced ER stress in mice.** Liver H&E staining **(A)** and its histological score **(B)** in tunicamycin-induced ER stress. Original magnification, x100 and x200. **C,** Western blot analysis of HSF2BP and GRP78 in tunicamycin-induced ER stress. Quantitative analysis of GRP78 **(D)** and HSF2BP **(E)** in tunicamycin-induced ER stress. Results are expressed as mean ± SE (n = 4-6/group) and compared by one-way ANOVA. * p < 0.05.

**
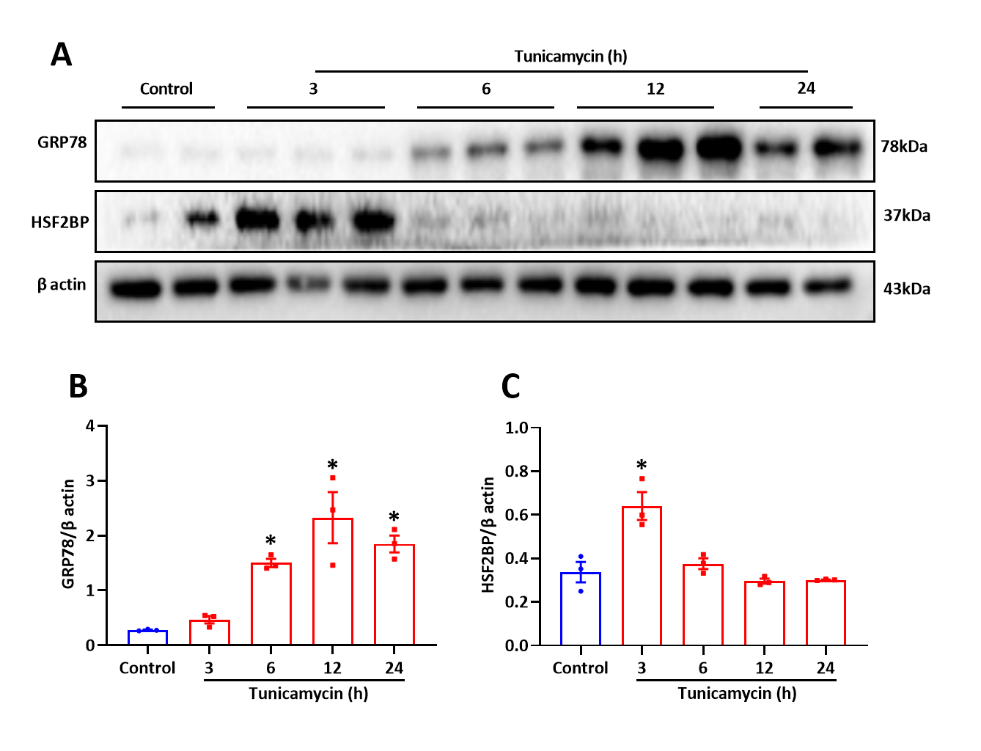
**

**Figure S2. HSF2BP expression is increased after tunicamycin-induced ER stress in cultured hepatocytes. A,** Western blot analysis of HSF2BP and GRP78 in HL-7702 cells. Quantitative analysis of GRP78 **(B)** and HSF2BP **(C)** in HL-7702 cells. Results are expressed as mean ± SE (n = 3/group) and compared by one-way ANOVA. * p < 0.05.


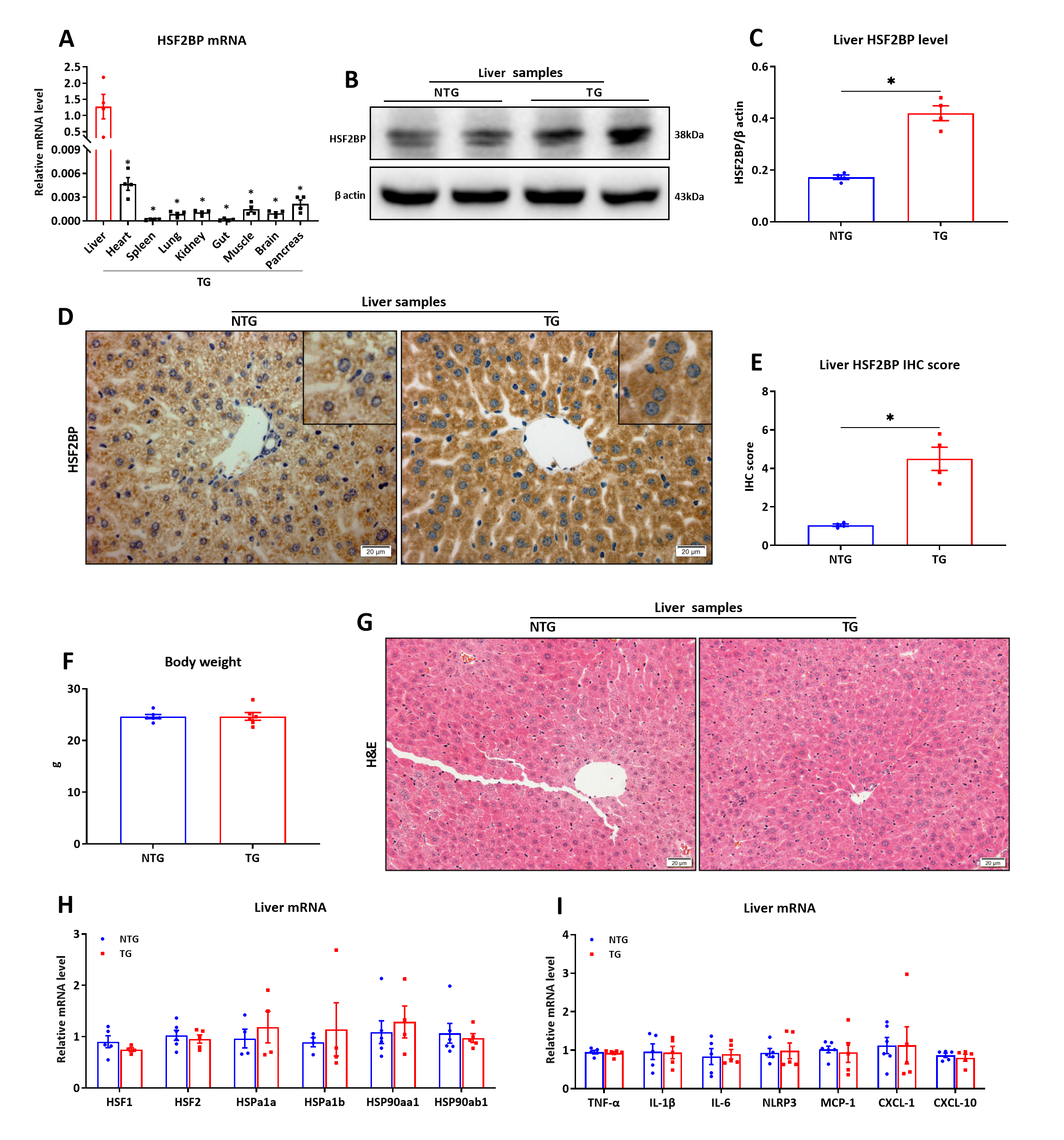


**Figure S3. The establishment of hepatocyte-specific HSF2BP transgenic mice. A,** The HSF2BP mRNA levels in various organs of HSF2BP transgenic (TG) mice. Western blot analysis of HSF2BP **(B)** and its quantitative analysis **(C)** in liver of hepatocyte-specific HSF2BP-TG and NTG mice. The immunohistochemical staining of HSF2BP **(D)** and its IHC score **(E)** in liver of HSF2BP-TG and NTG mice. Original magnification, x400. **F,** Body weight of HSF2BP-TG and NTG mice. **G,** The H&E staining in liver of HSF2BP-TG and NTG mice. Original magnification, x400. **H,** The levels of HSFs (heat shock factors) and HSPs (heat shock proteins) in liver of HSF2BP-TG and NTG mice. **I,** The levels of inflammatory factors in liver of HSF2BP-TG and NTG mice. Results are expressed as mean ± SE (n = 4-6/group) and compared by t-test or one-way ANOVA. * p < 0.05.


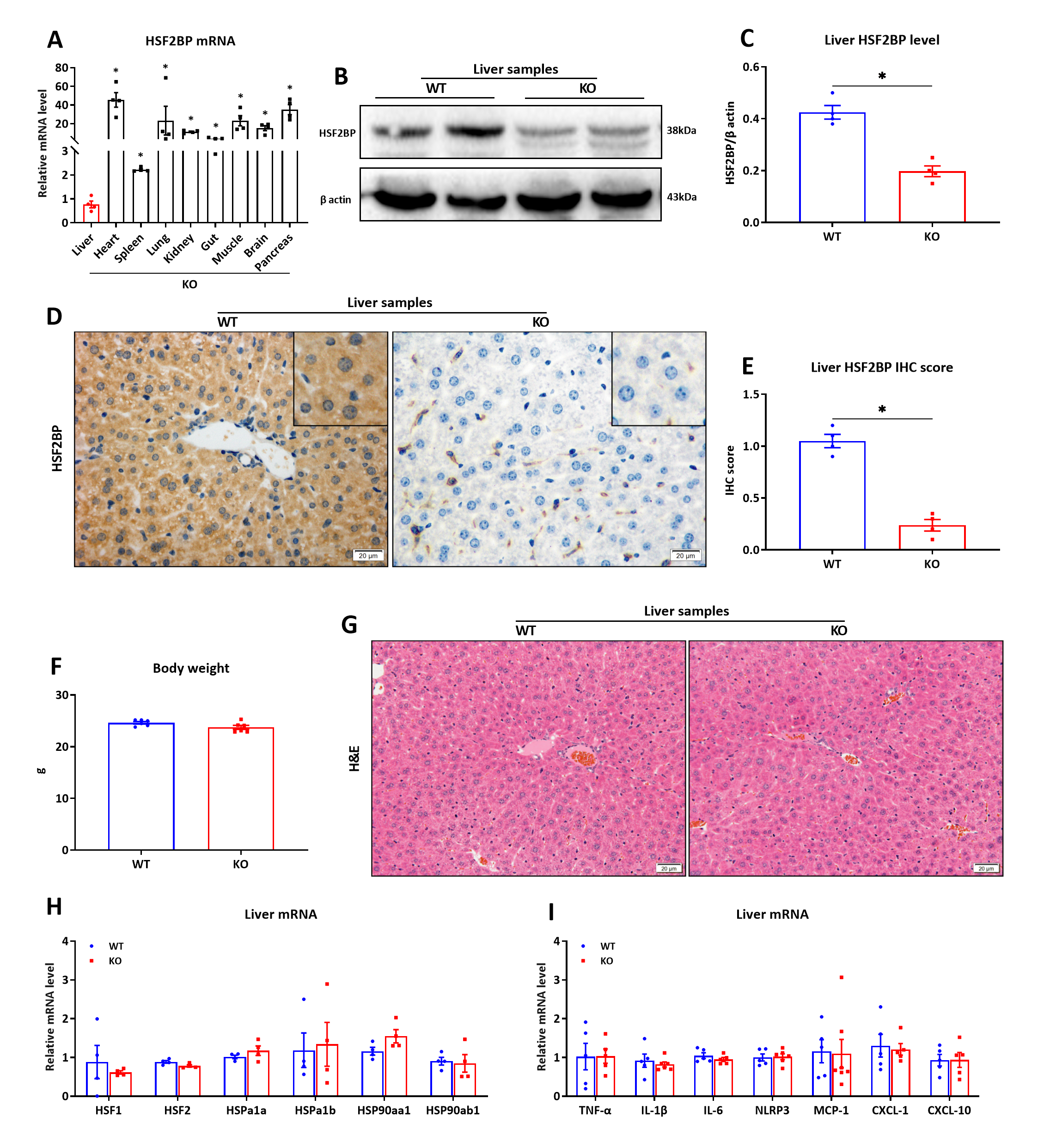


**Figure S4. The establishment of hepatocyte-specific HSF2BP knockout mice. A,** The HSF2BP mRNA levels in various organs of HSF2BP knockout (KO) mice. Western blot analysis of HSF2BP **(B)** and its quantitative analysis **(C)** in liver of HSF2BP-KO and WT mice. The immunohistochemical staining of HSF2BP **(D)** and its IHC score **(E)** in liver of HSF2BP-KO and WT mice. Original magnification, x400. **F,** Body weight of HSF2BP-KO and WT mice. **G,** The H&E staining in liver of HSF2BP-KO and WT mice. Original magnification, x400. **H,** The levels of HSFs (heat shock factors) and HSPs (heat shock proteins) in liver of HSF2BP-KO and WT mice. **I,** The levels of inflammatory factors in liver of HSF2BP-KO and WT mice. Results are expressed as mean ± SE (n = 4-6/group) and compared by t-test or one-way ANOVA. * p < 0.05.


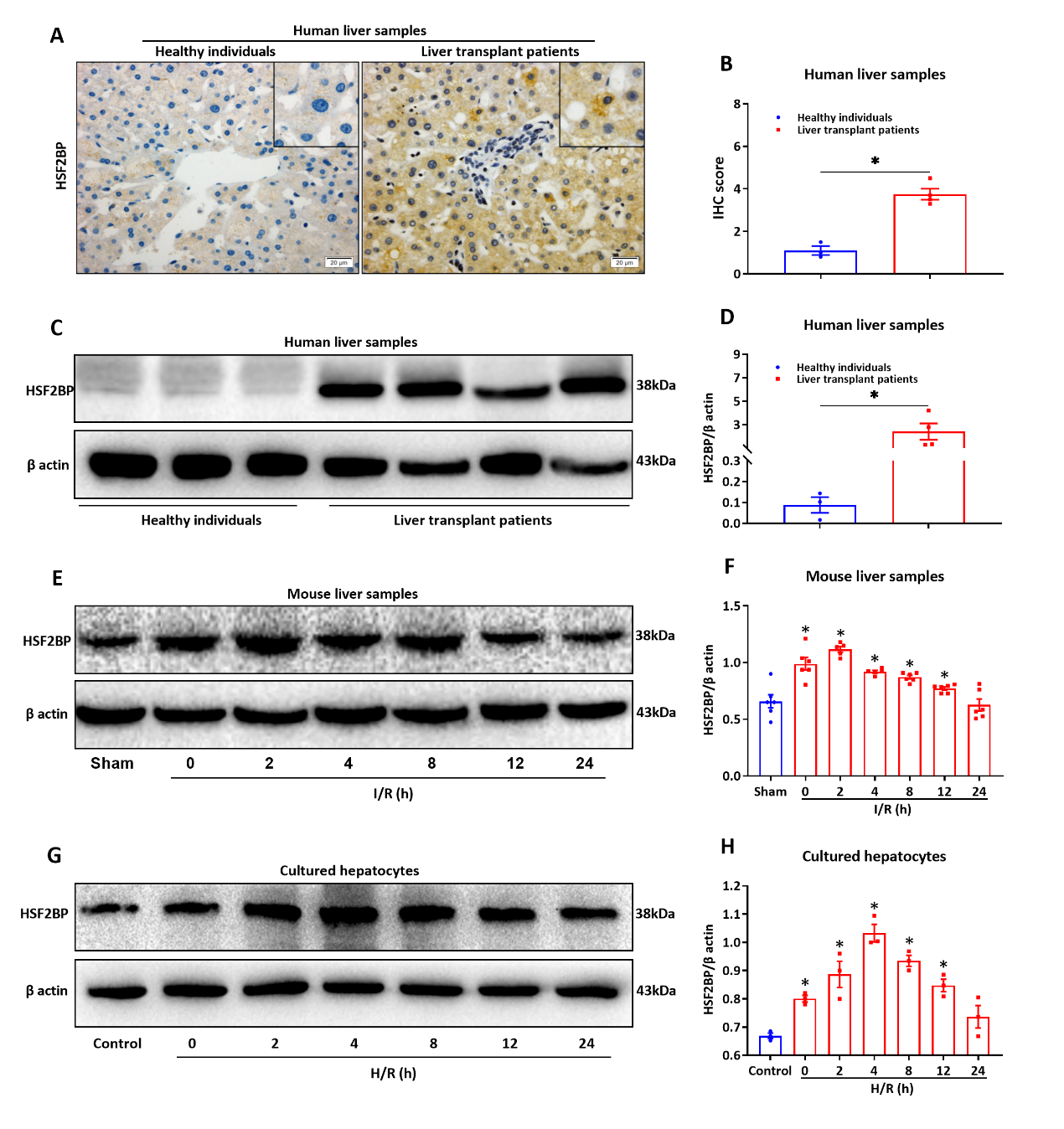


**Figure S5. HSF2BP is upregulated in hepatic I/R injury.** Liver immunohistochemical staining of HSF2BP **(A)** and its IHCscore **(B)** in liver transplant patients and healthy individuals. Western blot analysis of HSF2BP **(C)** and its quantitative result **(D)** in human livers of liver transplant patients (n = 4) and healthy individuals (n = 3). Western blot analysis of HSF2BP **(E)** and its quantitative result **(F)** in mouse livers subjected to ischemia and reperfusion (n = 4-6/group). Western blot analysis of HSF2BP **(G)** and its quantitative result **(H)** in cultured hepatocytes under H/R condition (n = 3/group). Results are expressed as mean ± SE and compared by t-test or one-way ANOVA. * p < 0.05 versus sham mice or control group.


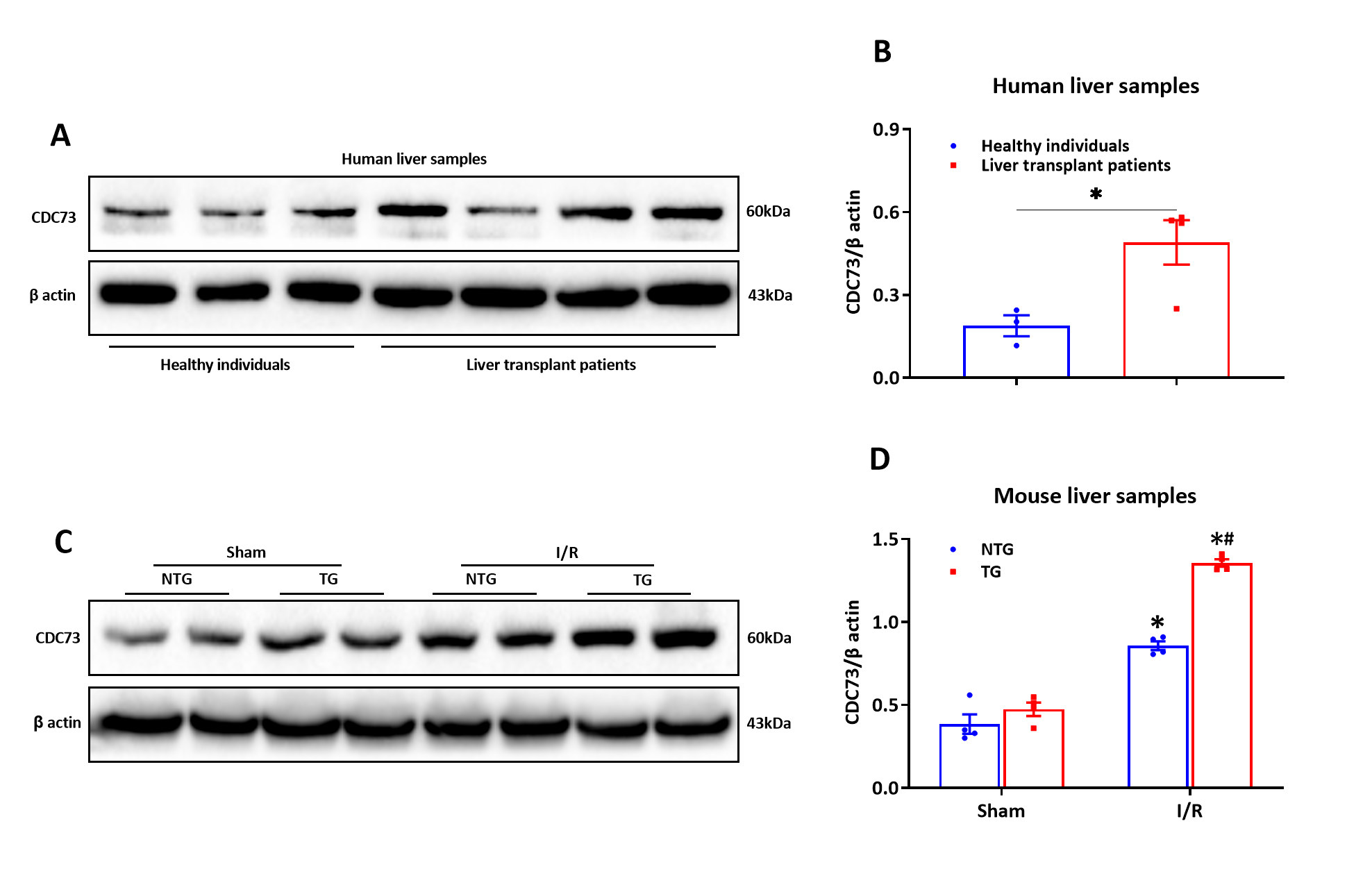


**Figure S6. CDC73 expression levels are increased in liver transplant patients and hepatic I/R mice.** Western blot analysis of CDC73 **(A)** and its quantitative analysis **(B)** in livers of liver transplant patients (n = 3) and healthy individuals (n = 4). Results are expressed as mean ± SE and compared by t-test. * p < 0.05. Western blot analysis of CDC73 **(C)** and its quantitative analysis **(D)** in mouse livers. Results are expressed as mean ± SE (n = 4) and compared by one-way ANOVA. * p < 0.05 versus sham mice, # p < 0.05 versus NTG mice.

**
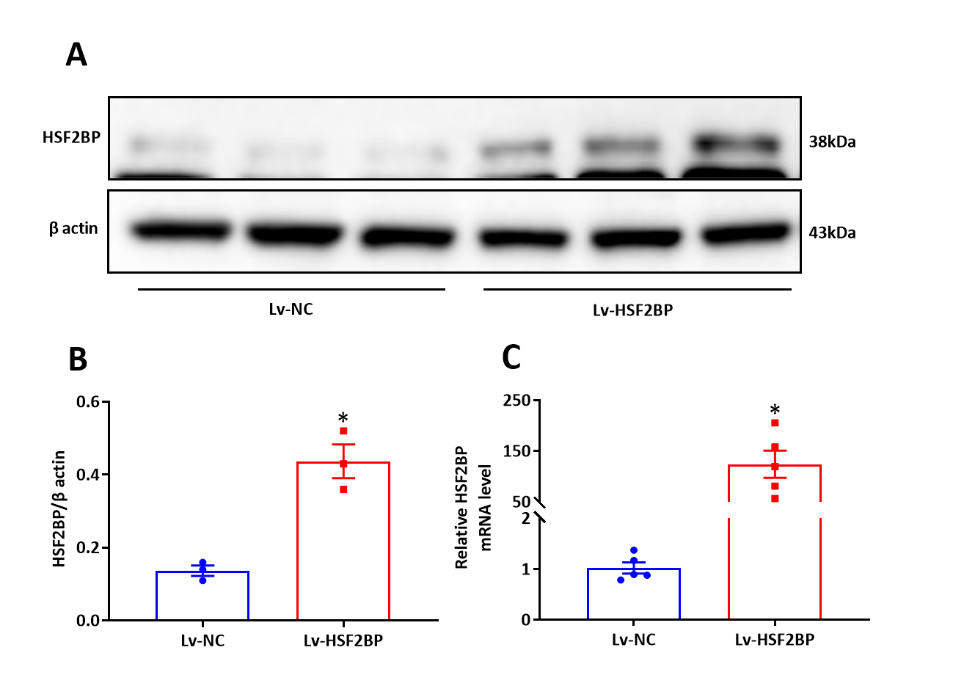
**

**Figure S7. HSF2BP expression is increased after transfection with HSF2BP overexpressed lentivirus in cultured hepatocytes.** Western blot analysis of HSF2BP **(A)** and its quantitative analysis **(B)** after transfection with Lv-HSF2BP in HL-7702 cells. **C,** The HSF2BP mRNA level after transfection with Lv-HSF2BP in HL-7702 cells. Results are expressed as mean ± SE (n = 3-5/group) and compared by t-test. * p < 0.05.


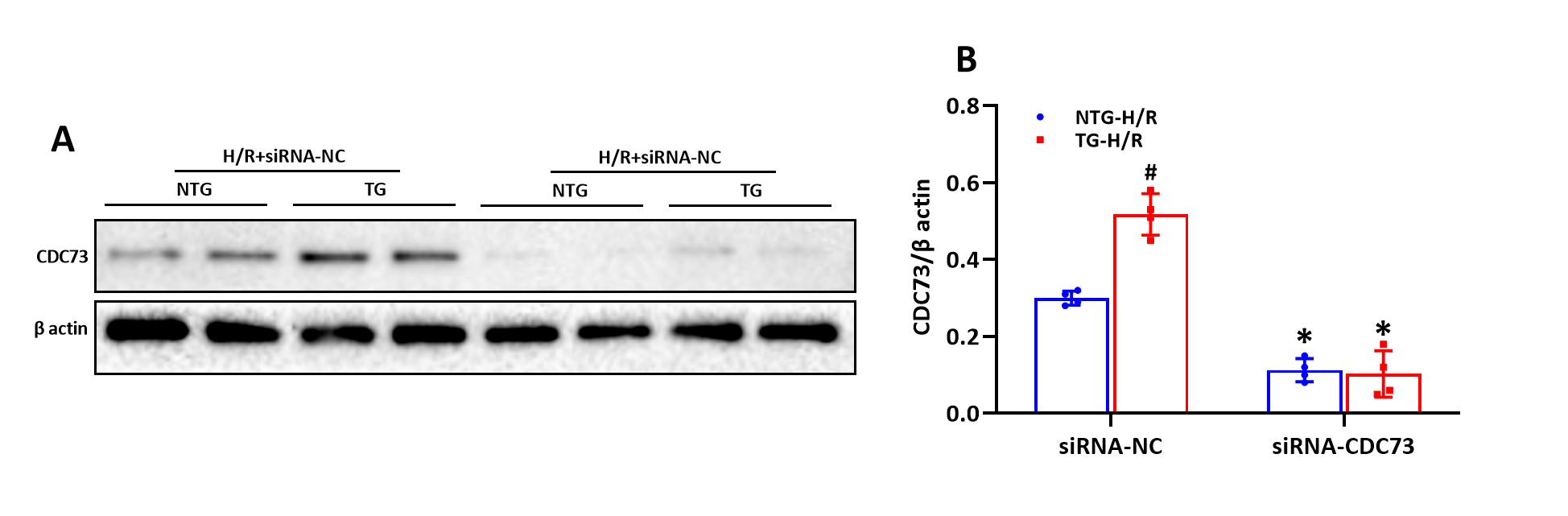


**Figure S8. CDC73 expression is knocked down after transfection with siRNA-CDC73 in primary hepatocytes.** Western blot analysis of CDC73 **(A)** and its quantitative analysis **(B)**after transfection with siRNA-CDC73 in primary hepatocytes. Results are expressed as mean ± SE (n = 4/group) and compared by one-way ANOVA. * p < 0.05 versus siRNA-NC group, # p < 0.05 versus NTG-H/R group.


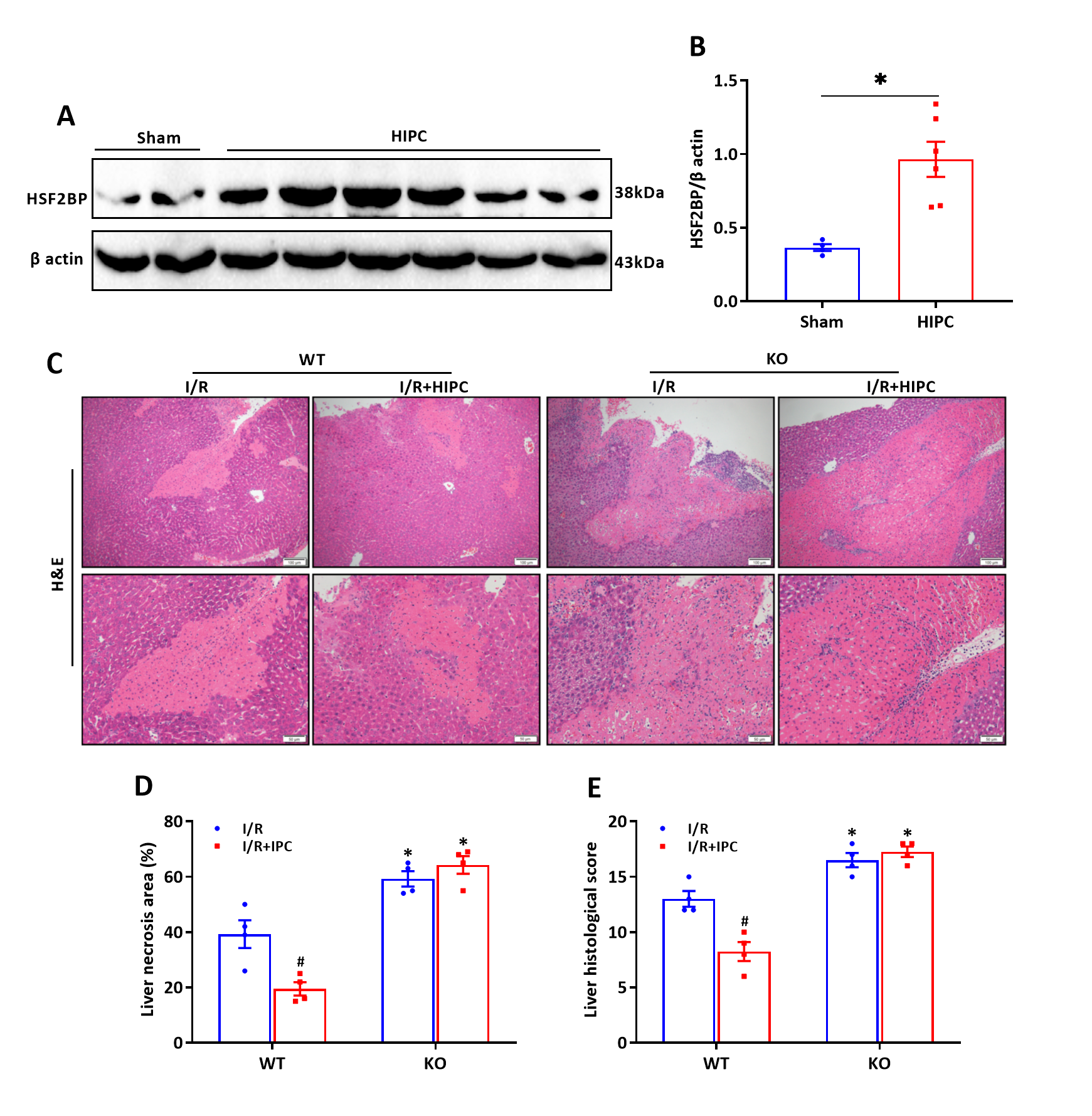


**Figure S9. Hepatic ischemic preconditioning (HIPC) up-regulated the expression of HSF2BP, and HSF2BP knockout eliminated HIPC’s hepatoprotective effect during hepatic I/R.** HIPC was induced by liver ischemia for 10 min, followed by 10 min of reperfusion. Western blot analysis of HSF2BP **(A)** and its quantitative analysis **(B)** after HIPC. Results are expressed as mean ± SE (n = 4-6/group) and compared by t-test. * p < 0.05 versus sham group. **C,** Liver H&E staining. Original magnification, x100 and x200. Liver necrosis area **(D)** and histological score **(E)**. Results are expressed as mean ± SE (n = 4/group) and compared by one-way ANOVA. * p < 0.05 versus WT mice, # p < 0.05 versus I/R group.
